# Mechanoregulative hydrogel facilitates rapid scarless healing by self-adaptive control of wound niche at different stages

**DOI:** 10.1126/sciadv.adv9895

**Published:** 2025-05-23

**Authors:** Haozhou Shu, Taotian Zhang, Yilin Jiang, Zhenkang Diao, Yani Xu, Jiaying Long, Tianyuan Chen, Mengxing Zhang, Zhirong Zhang, Junjie Chen, Shiqi Huang, Ling Zhang

**Affiliations:** ^1^ College of Polymer Science and Engineering, Sichuan University, Chengdu 610000, People’s Republic of China.; ^2^ Institute of Systems Epidemiology, West China School of Public Health and West China Fourth Hospital, Sichuan University, Chengdu 610041, People’s Republic of China.; ^3^ Key Laboratory of Drug-Targeting and Drug Delivery System of the Education Ministry, West China School of Pharmacy, Sichuan University, Chengdu, 610041, People’s Republic of China.; ^4^ Department of Burn and Plastic Surgery, West China Hospital, Sichuan University, Chengdu 610041, People’s Republic of China.

## Abstract

It is essential to spatiotemporally control the microniche of wound at different healing stages for rapid and scarless healing. Hence, a multifunctional soft hydrogel integrated with programmed drug release capability (MLVgel) was fabricated. MLVgel is adhesive and moldable with low friction response, which provides moisture and responses to an infectious environment to deliver bactericidal and antioxidant effects in rat bacterial infection and burn wound models. By release control, instant short-term inflammation suppression is combined with sustained mechano-transduction signal inhibition. This dynamically modulates immune responses and delays *En1*^+^ fibroblast activation via the YAP-TEAD pathway, ultimately facilitating scarless wound regeneration. Genomic analyses showed that the enhanced reepithelization and mechanoregulation by MLVgel during different wound phases are indispensable for its therapeutic outcomes. Last, MLVgel resulted in markedly improved healing in a pig mature scar model, which demonstrated its translation potential. Our results also verified the necessity of programed dynamic regulation in the healing process.

## Teaser

Dynamically controlled hydrogel promotes rapid scarless healing by regulating wound niche homeostasis at different stages.

## INTRODUCTION

In developed countries, more than 100 million individuals are affected by scarring (*1*), due to the lack of efficacious prevention and treatment strategies to reestablish matrix structure, regrow appendages, and restore mechanical robustness (*2*–*5*). The wound healing process mainly involves four interrelated and highly regulated stages: hemostasis, inflammation, proliferation, and remodeling (*6*). The regulation and interaction of fibroblasts, extracellular matrix (ECM), and immune microniche are important factors to maintain healing progression (*6*, *7*). Hence, abnormal ECM deposition owing to the loss of skin function, extended inflammatory period attributed to bacterial infection and other pathological factors, and abnormal fibroblast proliferation due to factors like mechanical force may ultimately result in scarring. This mainly involves the dysregulation of cell growth factors and proinflammatory factors, reactive oxygen species (ROS) disturbances (*8*), and increased intrinsic metabolic activity of the wound (*4*, *9*). Therefore, adjusting these factors during different healing stages and focusing on whole healing procedure are keys to achieve scarless wound healing.

Hydrogels attracted much interest as wound dressing due to their porous structure, excellent oxygen permeability, desirable drug loaded capacity, and robust moisturizing properties (*10*). In particular, in situ injectable hydrogels based on dynamic bonding offer additional benefits (*11*, *12*). However, the drug release pattern is uncontrollable owing to the weak structural force of injectable gel networks (*13*). Hence, it remains challenging to design a sustained release platform that can accommodate the dynamics of the microniche at different healing stages and regulate drug release behavior.

**Fig. 1.**
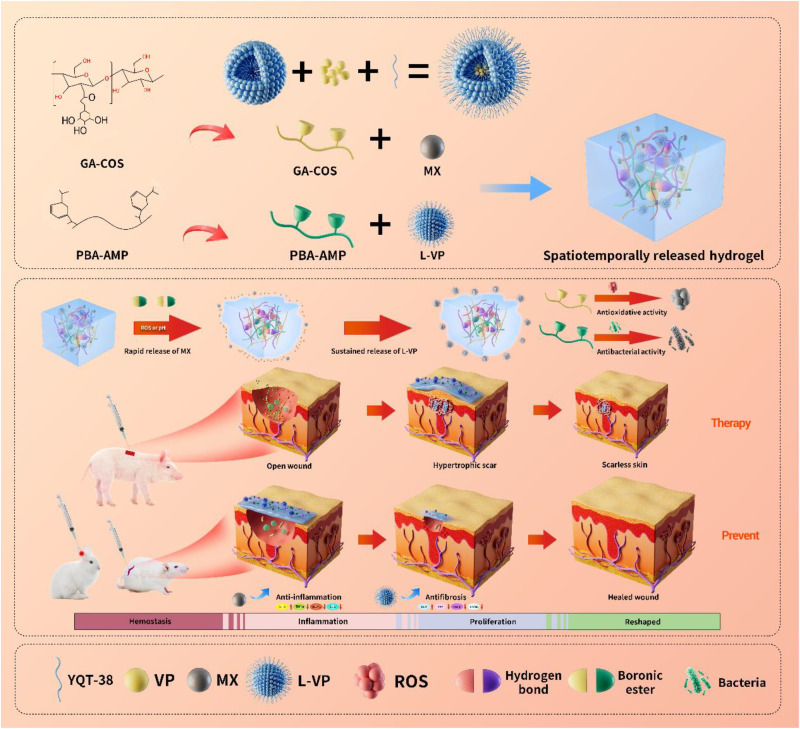
Diagram of the hydrogel preparation process and the mechanism of spatiotemporal control of wound microniche at different healing stages, which impedes scar formation.

In this study, inspired by the principles of infantile scarless wound healing and gel permeation chromatography, a multiresponsive injectable hydrogel for programmable controlled regulation of the inflammatory, proliferative, and remodeling stages of wounds was described (Fig. 1). This spatiotemporal release platform (MLVgel) is based on gallic acid (GA)–grafted chitosan oligosaccharide (COS, GA-COS) and 4-carboxy-3-fluorophenylboronic acid (PBA)–grafted antimicrobial peptide (AMPs, PBA-AMPs) (*14*). Meloxicam (MX), a nonsteroidal anti-inflammatory drug, was simultaneously carried into the hydrogel along with myo-fibroblast targeted liposomes encapsulating verteporfin (L-VP) (*15*). The targeted ability was endowed by an YQT-12 peptide. Furthermore, our methodology also resolves the ignored but important dilemma that VP applied in wound dressings may cause inevitable ROS damage under light conditions, along with a declined YES-associated protein (YAP) inhibition capacity by fluorescence resonance energy transfer (*homo*-FRET) at appropriate conjugate VP density in liposomes. (*16*).

The dynamic boronic ester bonds in MLVgel support three-dimensional grid structure and self-healing properties (*17*). Moreover, the GA-COS offers long-term tissue adhesion through spontaneous cross-linking reactions when exposed to physiological conditions (*18*). When applied to wounds, in response to the oxidizing acidic environment caused by inflammation and bacterial infection, boronic ester bond could break and free MX in a burst release fashion. Together with GA-COS and PBA-AMP, this leads to antibacterial effects, excess ROS reduction (*19*, *20*), and M1-to-M2 macrophage polarization induction, forwarding the phase transition from inflammation to proliferation (*21*–*24*). Subsequently, larger-sized liposomes exerted sustained and precise release of VP (*25*) to facilitate scar-free skin regeneration by inhibiting the YAP signaling and further blocking the activation of *Engrailed-1*(*En1^+^*) during wound healing (*26*, *27*), ultimately reducing the frequency of cell division during proliferation (*28*). Distinguished from existing wound healing strategies that mainly regulate wound healing in single stage, this platform focused on tracking the continuum of different wound healing stages. We highlighted the critical role of inflammatory and mechanistic regulation in both scar prevention and treatment, a concept that was further substantiated through tissue transparency imaging and comprehensive histological analysis. We raised up the concept that the hydrated lubrication layer of doped liposomes could inhibit wound reinfection.

The dual prophylactic and therapeutic properties in facilitating scarless wound regeneration were verified in various animal models, encompassing rat wound infection and burn models, as well as a rabbit ear hypertrophic scar (HS) model and a Bama miniature pig scar model. The immune infiltration was reduced after MLVgel treatment, with decreased profibrotic gene expression in fibroblasts and reestablished more normal matrix ultrastructure. These ultimately offered fast skin regeneration without scar. To sum up, our spatiotemporally released hydrogel had the potential to dynamically and precisely regulate the wound microniche and block scar formation, ultimately booting scarless wound healing.

## RESULTS

### Preparation and characterization of L-VP

**Fig. 2.**
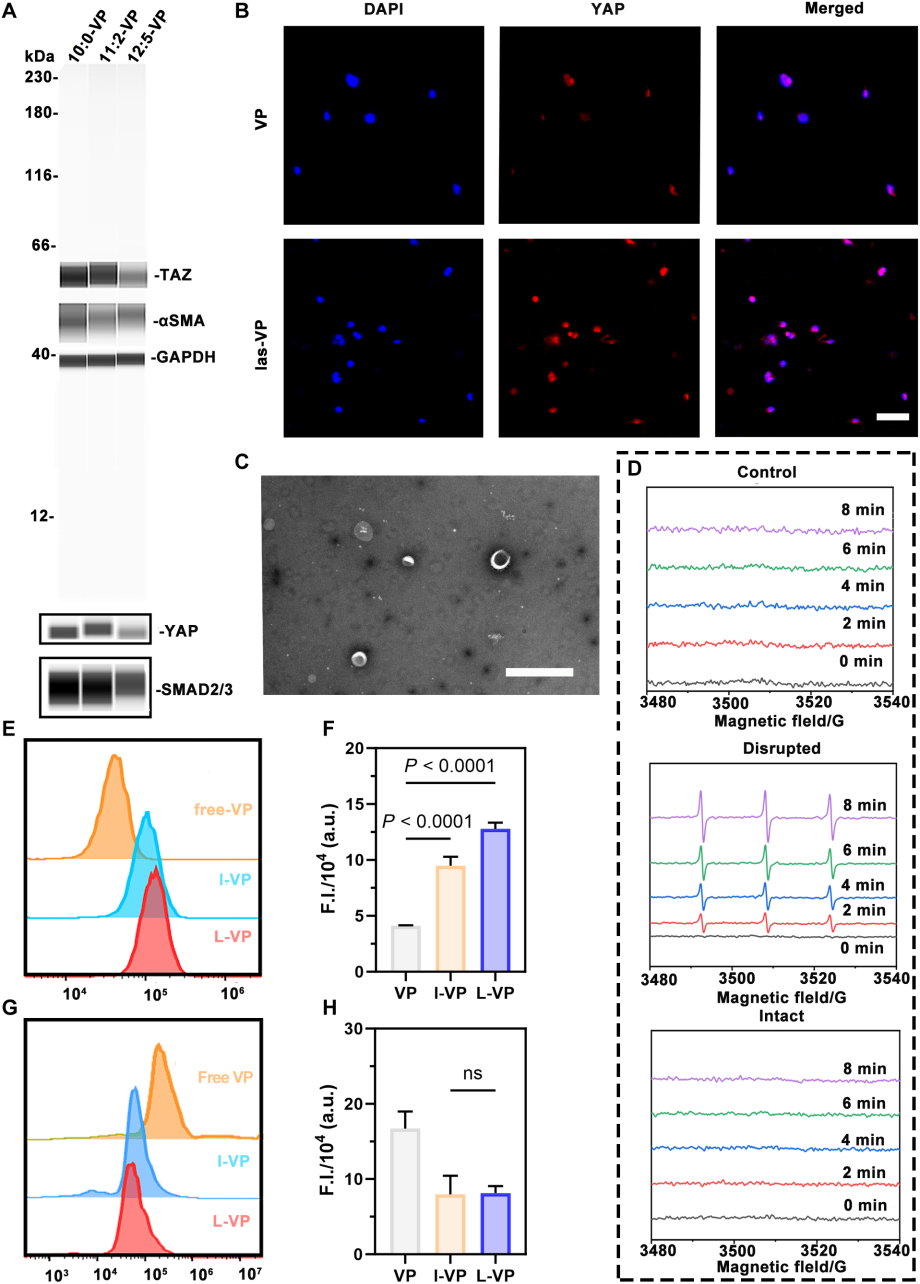
Characterization of VP and liposomes. (**A**) Protein levels of TAZ, YAP, α-SMA, and SMAD2/3 in L929 cells treated with different concentrations of VP as determined by Simple Western blot. (**B**) Immunofluorescence images of L929 fibroblast stained with YAP after treated for 24 hours. Scale bar, 5 μm. (**C**) TEM image of L-VP. Scale bar, 500 nm. (**D**) ESR absorption curve of intact and disrupted L-VP with a 660-nm laser in different time points. (**E**) Representative results of flow cytometry determination of VP, l-VP, and L-VP uptake capacity by L929 fibroblasts and (**F**) statistical analysis (*n* = 3). (**G**) Representative results of flow cytometry determination of VP, l-VP, and L-VP uptake capacity by RAW264.7 macrophages and (**H**) statistical analysis (*n* = 3). Data were presented as means ± SD, and statistical significance was analyzed via one-way analysis of variance (ANOVA) with Tukey’s multiple comparisons test. DAPI, 4′,6-diamidino-2-phenylindole; F.I., fluorescence intensity; a.u., arbitrary units; ns, not significant.

VP is a second-generation porphyrin photosensitizer approved by the Food and Drug Administration for photodynamic treatment of macular disease due to its ability to continuously release ROS under laser irradiation (*29*). In addition, it can inhibit YAP without light activation (*30*), thereby preventing *En-1* activation and promoting scarless wound regeneration (*26*). Simple Western blot assay was conducted to demonstrate its inhibitory effect on YAP/TAZ, drosophila mothers against decapentaplegic protein 2/3 (SMAD2/3), and α–smooth muscle actin (α-SMA) in fibroblasts (Fig. 2A and fig. S1) (*31*). However, the photosensitive effect of VP appeared to contradict wound repair (*32*), where it exhibited cytotoxicity at lower concentrations compared with MX (fig. S2, A and B) and alleviated an inhibitory effect on YAP protein (Fig. 2B) after full laser irradiation (las-VP). L-VP could resolve the dilemma, the targeted ability would be endowed by YQT-12 peptide decoration, and the peptide was coupled to liposomes through the reaction between the cysteine residue of YQT-12 and maleimide of DSPE-PEG2000-Mal.

The average sizes of the VP loaded liposomes (l-VP) and L-VP were 123.23 ± 1.37 nm and 125.06 ± 1.26 nm, the polymer dispersity index (PDI) of l-VP and L-VP were 0.286 ± 0.021 and 0.271 ± 0.005, and the zeta potentials of l-VP and L-VP were 7.06 ± 0.22 mV and 10.20 ± 0.08 mV, respectively. A transmission electron microscopy (TEM) image showed that the L-VP were roughly spherical (Fig. 2C) with uniform particle sizes. The photosensitive effect of highly concentrated VP was found to be turned off under the *homo*-FRET effect. Electron paramagnetic resonance (EPR) results showed that L-VP at the same concentration did not generate free radicals after 8 min of full laser activation (660 nm), compared to disrupted L-VP (Fig. 2D). This conclusion was consistent with the fluorescence images (fig. S3), demonstrating the feasibility of using lipid-based nanoparticles to delay the VP phototoxicity. Flow cytometry results (Fig. 2, E and F) and confocal laser scanning microscopy (CLSM) imaging (fig. S4, A and B) showed that the uptake efficiency of VP by fibroblasts was notably increased in the L-VP group compared to the free VP group. Macrophage uptake showed the opposite effect (Fig. 2, G and H), which may be attributed to the differences in nanoparticle properties that macrophages and fibroblasts tend to uptake. Liposomes with higher membrane fluidity were easily deformed and therefore energetically unfavorable for macrophage uptake. Macrophages could endocytose nanoparticles via scavenger receptors, which display remarkable preference for negative charges. These specificities may lead to the reduced uptake of positively charged liposomes l-VP and L-VP (*33*–*36*).

### Synthesis and characterization of MLVgel

**Fig. 3.**
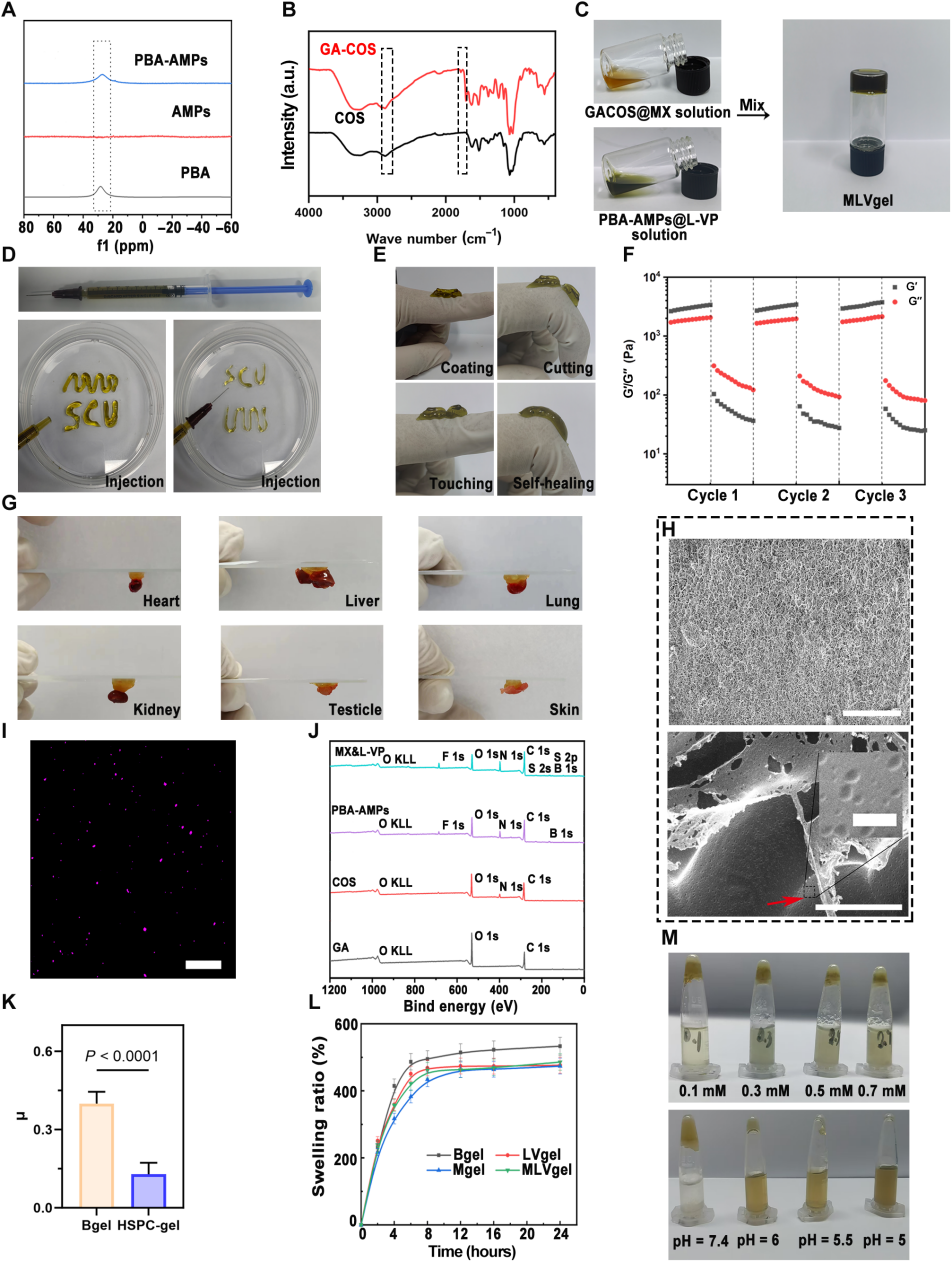
Characterization of hydrogel. (**A**) ^11^B NMR spectra of AMPs, PBA, and PBA-AMPS. (**B**) Fourier transform infrared spectra of COS and GA-COS powders. (**C**) Representative image of hydrogel formation. (**D**) Injectable and (**E**) self-healing property of the hydrogel. (**F**) Continuous step-strain experiments conducted on the hydrogels, subjecting them to repeated deformations of 1 and 300% strain. (**G**) Photographs depicting the adhesion of hydrogels to various biological tissues. (**H**) Cryo-SEM representative image of hydrogel, with a red arrow indicating the presence of liposomes exposed at the surface of the hydrogel’s frozen cracks. Scale bars range from large to small: 50 μm, 3 μm, and 200 nm, respectively. (**I**) Representative CLSM images of hydrogels containing DiD-labeled liposomes (purple). Scale bar, 2 μm. (**J**) Full spectrum of XPS images. (**K**) Coefficient of sliding friction between stainless steel balls and hydrogels with or without liposomes (*n* = 5). Normal load, *F_n_*: 0.1 N. (**L**) Swelling ratios of hydrogels (*n* = 3). (**M**) Responsive behavior images of hydrogel under different H_2_O_2_ concentrations (0.1, 0.3, 0.5, and 0.7 mM) and different pH (7.4, 6.5, 6, and 5.5) values. Data were presented as means ± SD, and statistical significance was analyzed via *t* test.

Given the highly dynamic nature of wound healing and the complexity of the microniche, effectively preserving and protecting drug capsules in wounds have become a notable challenge in delivering wound repair carriers. Injectable hydrogels based on dynamic boron esters can enable strong adhesion of biomaterials to tissues and promote wound healing (*37*, *38*). To obtain the MLVgel, we first prepared PBA-AMPs and GA-COS. Considering that PBA could form reversible boronic ester bonds with diols under neutral or weakly alkaline conditions, the amino group of the lysine side chain in AMPs (fig. S5) was coupled with PBA through an amidation reaction (*39*). ^11^B nuclear magnetic resonance (NMR) spectrogram demonstrated that PBA was successfully coupled to the AMPs (Fig. 3A), where the phenylboric acid peaks were exhibited at 20 parts per million (ppm) (*40*), while no typical signal was detected in AMPs. In addition, the same grafting scheme was used for COS and GA. COS was selected owing to its eminent biocompatibility and antibacterial properties. Unlike chitosan, COS can be dissolved in ultrapure water. Meanwhile, GA not only has a high affinity for PBA (*40*) but also has excellent antioxidant and anti-inflammatory effects. ^1^H NMR results demonstrated the successful coupling of GA to COS (fig. S6), with a new proton peak observed at 6.90 ppm attributed to the phenol group of GA, in stark contrast to the initial COS results. The Fourier transform infrared results of GA-COS and COS (Fig. 3B) were consistent with this observation (*41*). In GA-COS, it was observed that the CO-NH structure’s vibration peak at 1617 cm^−1^ belonging to the amide I band shifted to a higher-frequency region indicating a change in the amide component unit within chitosan molecular chain due to amidation reaction. Furthermore, amidation reaction weakened the stretching vibration peak belonging to O─H and N─H bonds at 2800 to 3500 cm^−1^ while enhancing O─H absorption peak by introducing the phenol group. These abovementioned variations suggested that GA was introduced into COS through amidation reaction. In addition, a new increased peak corresponding to carbonyl (C═O) stretching in the ester group was observed at 1746 cm^−1^, confirming the formation of an ester bond between the carboxyl group of GA and the hydroxyl group of COS.

Representative images illustrating the gelation process were presented in Fig. 3C. Injectable hydrogels render a promising utility for promoting in situ tissue repair, and soft cross-linking promotes cell infiltration and healing and plays a positive role in the regulation of macrophages and fibroblasts (*42*). The written letters were formed through successive injection (Fig. 3D), indicating the superior injectability of MLVgel. Then, it was injected on the finger and cut into two segments, as depicted in Fig. 3E. MLVgel demonstrated the self-repair capacity and could withstand a certain bending force. These findings were consistent with the results of the cyclic strain experiment on hydrogel rheology (Fig. 3F). Furthermore, we demonstrated that the hydrogel exhibited excellent bioadhesion (Fig. 3G) and material adhesion properties (fig. S7A). It was observed that under normal activity conditions, MLVgel remained on the arm for an extended period of time without requiring additional immobilization (fig. S7B), which may be ascribed to the existence of GA-COS.

The dispersion and stability of liposomes in hydrogels are crucial factors (*43*). Briefly, after 30 days of storage at 4°C, the representative cryo–scanning electron microscopy (cryo-SEM) results were presented in Fig. 3H and showed that the MLVgel exhibited a three-dimensional pore structure, and the spheres indicated by the red arrow represented well-preserved liposomes, with a particle size of approximately 100 nm and uniform dispersion. The CLSM results of hydrogels containing DiD-loaded liposomes (L-DiD@gel) further confirmed the uniform dispersion of liposomes in gels (Fig. 3I and fig. S8) and also demonstrated the effectiveness of liposome gel coencapsulation strategy to reduce drug leakage and improve long-term storage. Furthermore, the results of x-ray photoelectron spectroscopy (XPS) and SEM–energy dispersive spectroscopy analysis consistently revealed a homogeneous distribution of B, C, N, O, and other elements in MLVgel (Fig. 3J and fig. S9). The F 1s presence in XPS results of MLVgel proved successful coupling of PBA. In addition, the S 2s and S 2p presence in MLVgel may originate from MX.

Subsequently, we investigated the hydration lubrication effect based on liposome doping, which can reduce the adherence ability of bacteria by trapping water molecules through strong attraction and correspondingly forming a stable hydration layer (*44*, *45*). As shown in friction experiment results (Fig. 3K and fig. S10), Lip@gel exhibited a strong reduction in friction coefficient and maintained stable wear resistance under linear reciprocating friction conditions compared to Lip-free gel (*43*), highlighting the robust hydration lubrication effect of these lipid-containing hydrogels and foreshadowing their bioprotective properties.

The swelling behavior of hydrogel is directly correlated to the absorption of tissue exudates and drug release efficiency (*46*). Here, we found that MLVgel maintained high water absorption within 8 hours and reached swelling equilibrium after 12 hours with a swelling ratio of 4.5 to 5.2 (Fig. 3L), demonstrating the excellent moisture retention ability and structural stability of hydrogels. Continuous degradation behavior was also observed in different hydrogels (fig. S11A), indicating their desirable biodegradability. To investigate the responsiveness of MLVgel under different physiological conditions, the hydrogels were treated in different pH values or different concentrations of H_2_O_2_ at 37°C for 24 hours. It was observed that MLVgel exhibited decreased stability in simulated inflammation niches (Fig. 3M), demonstrating the potential ability to release therapeutic agents in wound sites owing to the existence of boronic ester bonds. To initially confirm the speculation, the in vitro release behavior of MX and VP loaded in different spatial positions was determined by liquid chromatography–mass spectrometry (LC-MS), where the precise spatiotemporal release behavior of L-VP and MX was regulated by cargoes and microniche (fig. S11, B and C). In the wound stimulated niche rather than physiological situations, the hydrogels exhibited accelerated MX and L-VP release; however, the release pattern of VP loaded in liposomes was absolutely slower than that of free MX during the first 2 days, suggesting that the spatiotemporal delivery behavior of hydrogels was highly consistent with the programmed wound healing process.

Last, the dynamic rheological behavior of MLVgel was investigated. The viscosity and elastic properties under different stresses and strains were represented by storage modulus (G′) and loss modulus (G″) (*47*). As depicted in fig. S11D, G′ was consistently greater than G″ for all hydrogels within the range of 0.1 to 100 rad s^−1^, indicating that the formation of a hydrogel elastic network and the existence of MX and L-VP would not interrupt the rheological properties. Moreover, the gelation time was determined as the point where G′ and G″ intersected using a rheometer at room temperature and under low stress and strain conditions (fig. S11E) and was found to be less than 2 min for all hydrogels.

### In vitro therapeutic effects of MLVgel

**Fig. 4.**
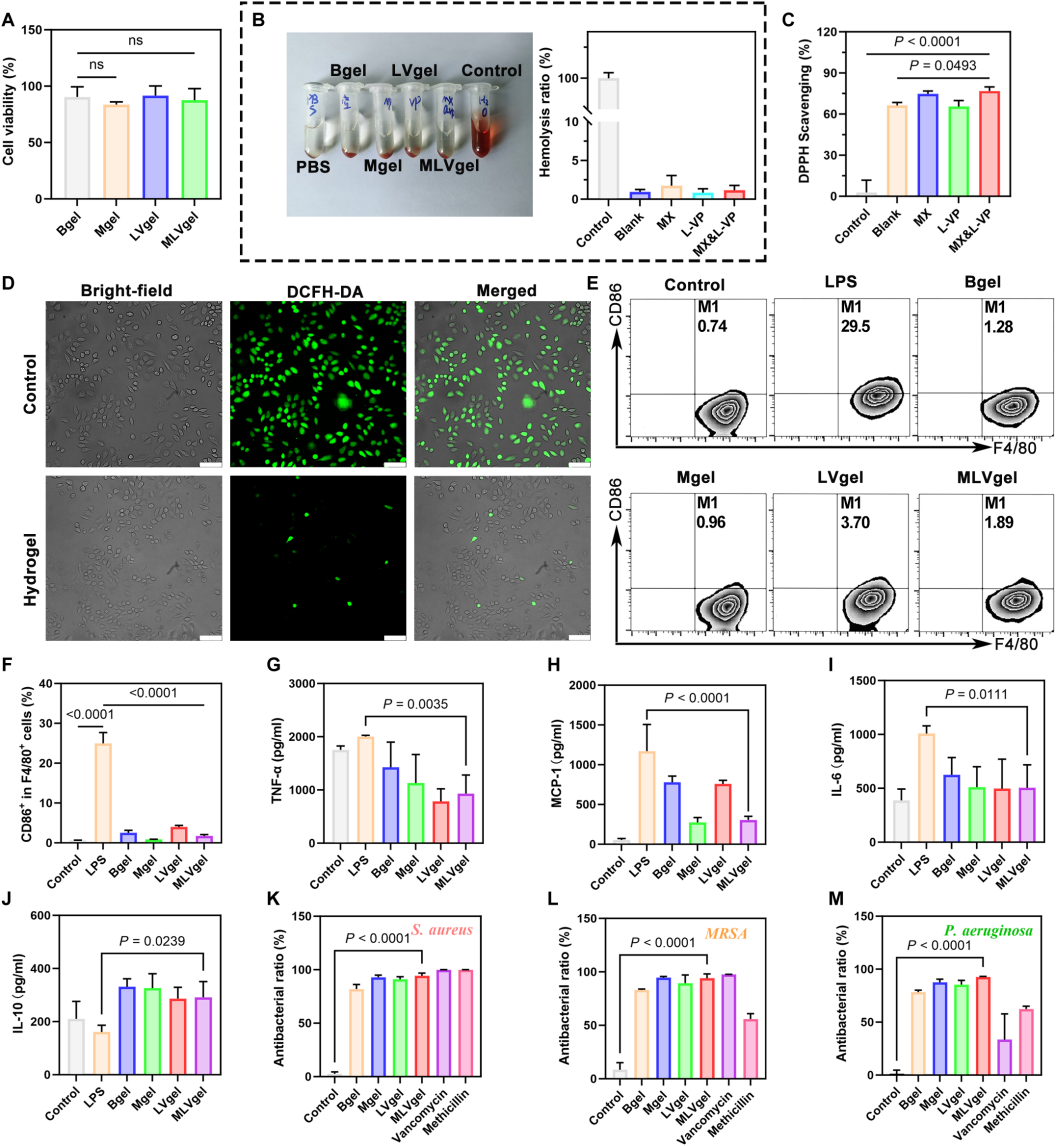
Effects of hydrogels in vitro. (**A**) Cytotoxicity of different hydrogels on L929 fibroblasts for 3 days (*n* = 4). (**B**) Image of hemolysis test and statistical analysis (*n* = 6). (**C**) DPPH radical scavenging ability of the hydrogels (*n* = 3). (**D**) Fluorescence images of DCFH-DA in H_2_O_2_-treated L929 cells. Scale bars, 50 μm. (**E**) Flow cytometry results and (**F**) statistical analysis of RAW264.7 macrophage polarization after treated with different hydrogels (*n* = 3). (**G** to **J**) Relative quantitative analysis of the representative cytokines detecting by ELISA (*n* = 3). Quantitative analysis of the antibacterial ratio of hydrogels against (**K**) *S. aureus*, (**L**) *MRSA*, and (**M**) *P. aeruginosa* via the absorbance method (*n* = 3). Data are presented as means ± SD, and statistical significance was analyzed via one-way ANOVA with Tukey’s multiple comparisons test.

Clinical wound dressings usually require hydrogels with excellent biocompatibility (*48*). Hence, the biocompatibility of MLVgel was evaluated in vitro by conducting cell viability and hemolysis assays. Initially, after coincubation L929 cells with different hydrogel groups for 3 days, no significant changes in cell viability were found compared to the control group (Fig. 4A), indicating their favorable biocompatibility. Considering that wound dressings inevitably come into contact with blood, the hemolysis test was further used to assess biocompatibility. The results indicated that none of the hydrogels incubated with red blood cells (RBCs) exhibited noticeable hemolysis (Fig. 4B), and the hemolysis rate of all groups was found to be less than the 5% threshold required for biomaterials (*49*).

During the inflammatory period of wound healing, the overexpression of inflammatory factors and an increase in ROS levels may be caused by bacterial infection and considered to impede wound healing process and boost scar formation (*50*, *51*). DPPH free radical detection in vitro and the results demonstrated that the free radical scavenging efficiency of all hydrogels exceeded 65% (Fig. 4C), indicating a strong antioxidant effect of MLVgel. Subsequently, L929 cells were treated with H_2_O_2_, and the DCFH-DA fluorescent signal was detected by a fluorescence microscope to confirm ROS levels. As shown in Fig. 4D and fig. S12, the decreased signal shown in the MLVgel-treated group suggested successful inhibition of cell oxidative stress.

Worthily, considering that macrophages play a crucial role in regulation of the inflammatory stage, the anti-inflammatory effect of MLVgel was further assessed by incubating lipopolysaccharide (LPS)–stimulated RAW264.7 cells with hydrogel extract for 24 hours. The results of flow cytometry demonstrated that MLVgel (1.89%) markedly inhibited macrophage polarization to M1 phenotype compared to the LPS-induced untreated group (29.5%), thus decreasing inflammation levels (Fig. 4, E and F). Subsequently, the quantification of immune-regulated cytokines was carried out via enzyme-linked immunosorbent assay (ELISA) (Fig. 4, G to J). We found that MLVgel effectively down-regulated the expression of tumor necrosis factor–α (TNF-α), MCP-1, and interleukin-6 (IL-6) while up-regulating the expression of IL-10 compared to the LPS-induced untreated group, and this finding was further confirmed by cross-validation experiments with quantitative reverse transcription polymerase chain reaction (fig. S13).

We also validated the antibacterial efficacy of hydrogels against bacteria commonly found in wound infection. The results indicated that the hydrogel exhibited consistently robust antibacterial properties against Gram-negative bacteria *Pseudomonas aeruginosa*, Gram-positive bacteria *Staphylococcus aureus*, and methicillin-resistant *S. aureus* (*MRSA*) (Fig. 4, K to M, and fig. S14), where broad-spectrum antibiotics methicillin and vancomycin were used as positive control groups. Results proved that MLVgel could resolve the dilemma of the poor inhibition effect of methicillin on *MRSA* and the fluctuating inhibition effect of both antibiotics on *P. aeruginosa*.

### MLVgel enhanced scarless wound healing in rat bacterial infection wound and burn wound model

**Fig. 5.**
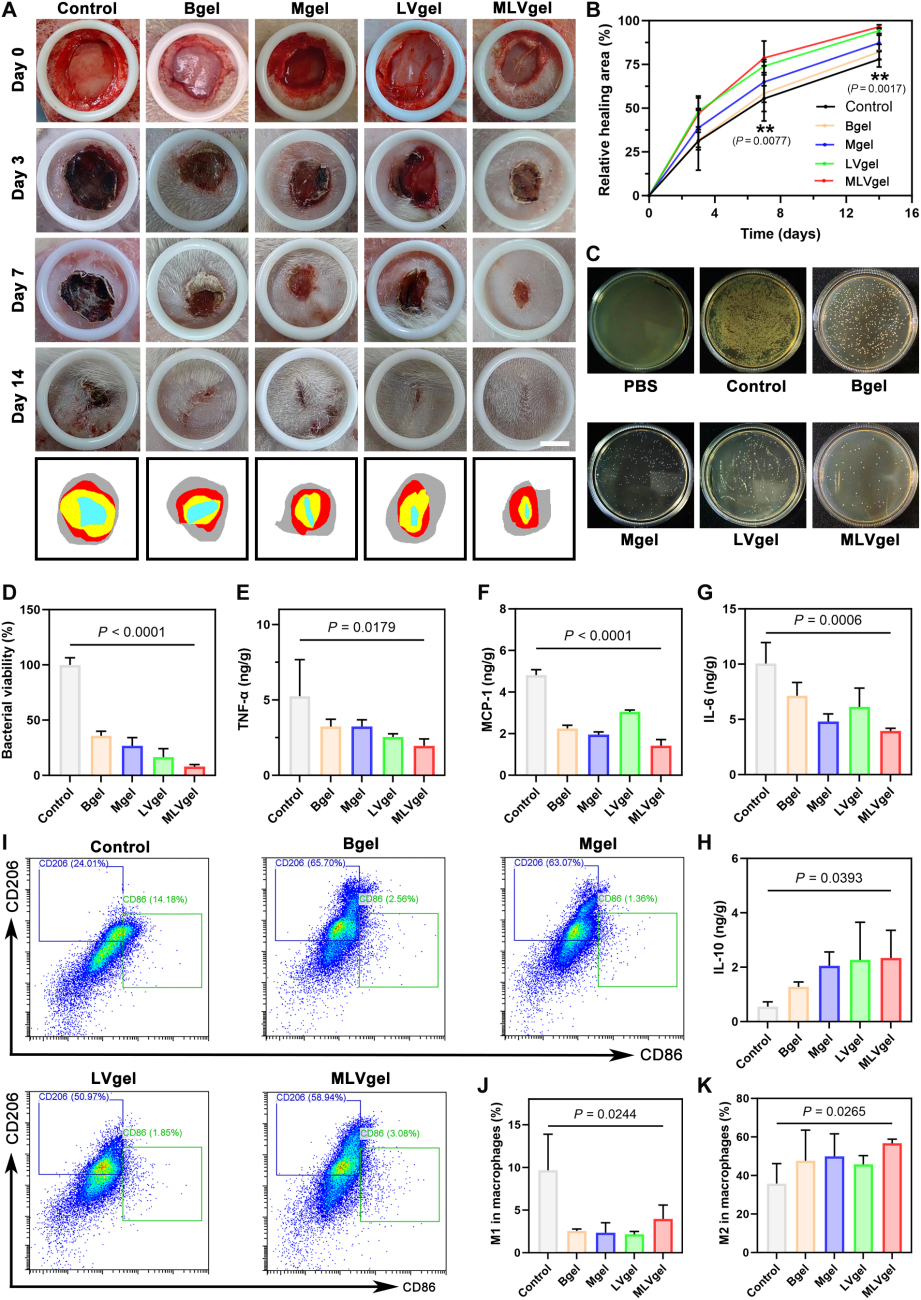
In vivo wound healing effect in rat model of whole-cortex infection. (**A**) Wound photographs of SD rats in various treatment groups at different time points. Scale bar, 3 mm. (**B**) Quantitative analysis of the relative wound healing rate at different times (*n* = 4). (**C**) Photographs of viable bacteria on the LB agar plates after 3 days of various treatments and (**D**) statistical analysis (*n* = 3). ELISA detected the concentrations of (**E**) TNF-α, (**F**) MCP-1, (**G**) IL-6, and (**H**) IL-10 (*n* = 3). (**I**) Flow cytometry of wound macrophage typing in SD rats with different treatments and (**J** and **K**) statistical analysis (*n* = 3). Data are presented as means ± SD, and statistical significance was analyzed via one-way ANOVA with Tukey’s multiple comparisons test.

Encouraged by the abovementioned results, it is plausible to assume that MLVgel has the potential to enhance wound regeneration and allay scarring. We first created full-cortex wound on the back of rat and exposed it to *P. aeruginosa* for 24 hours to fabricate the rat bacterial infection wound model. Subsequently, the rats were randomly divided into five groups and treated with phosphate-buffered saline (PBS; control), no drug loaded hydrogel (Bgel), only MX (Mgel), only L-VP (LVgel) and MLVgel, respectively. The wound area was measured and photographed on days 0, 3, 7, and 14. As depicted in Fig. 5 (A and B), MLVgel- and LVgel-treated groups exhibited accelerated wound healing and expedited wound closure, with substantially better wound closure ratios than the control group on days 3 (49.4%) and 7 (73.6%), respectively. In vivo antibacterial properties of different hydrogel-treated groups were assessed by collecting wound tissues on day 3 (Fig. 5, C and D). It could be found that wound sites treated with MLVgel showed a reduction in the number of bacterial colonies with the lowest bacterial viability percentage of almost 90% and indicated effective inhibition of bacterial infection, which was mainly attributed to the antibacterial properties of AMPs as well as its hydration and lubrication effect conferred by the phospholipid bilayer. In addition, the expression levels of various inflammatory factors in the same wound tissues including IL-6, TNF-α, MCP-1, and IL-10 were measured by ELISA (Fig. 5, E to H), and the polarization of macrophages was confirmed by flow cytometry (Fig. 5, I to K, and fig. S15). The results indicated that owing to the fracture response of hydrogels and the high efficiency of MX release, MLVgel could effectively decrease the expression of proinflammatory factors and increase the expression of anti-inflammatory factors during the inflammatory period to regulate wound microniche. In addition, the results of macrophage phenotype analysis indicate that after MLVgel treatment, the levels of the M1 phenotype were reduced; however, a slight increase in the M2 phenotype, which facilitates progression to the next stage of recovery, in the wound healing process was definitely restrained by the treatment of MLVgel. Simultaneously, the levels of various immune cells such as lymphocytes, white blood cells, and neutrophils were decreased in MLVgel-treated rats (fig. S16).

Furthermore, wound tissues were collected on day 7 and stained with immunofluorescence. Integrated optical density analysis revealed that rats treated with MLVgel mitigated the levels of the M1 phenotype marker CD86 compared to the control group (fig. S17, A and B), with the increased levels of the M2 phenotype marker CD206 (fig. S17C). In addition, levels of the proinflammatory factor TNF-α were decreased after MLVgel treatment (fig. S17D), indicating reduced wound inflammation in rats. The up-regulated levels of the proangiogenic factor platelet endothelial cell adhesion molecule 1 (CD31) and the down-regulated levels of activated/profibrotic myofibroblast marker α-SMA (fig. S17, E and F) suggested that MLVgel had the potential for dynamically and precisely regulating the wound microniche and blocking scar formation ascribed to the addition of L-VP and MX, demonstrating the necessity of sequence regulation in promoting wound healing process.

Hematoxylin and eosin (H&E) and Masson staining could also be used to evaluate the effects of hydrogels on the wound healing process. Compared to the control group, the degree of immune infiltration was impeded after sequential release treatment, and more matrix ultrastructure was reestablished on day 7 (fig. S18A). After 14 days of treatment, the wounds treated with MLVgel yielded skin regeneration without scar, along with regular arrangement of collagens and recovery of functional hair follicles (fig. S18, A and B). Sirius Red staining was used to further analyze the proportion of different types of collagens in the wound, where the type I collagen (COL-I) often plays a major role in scar formation, with notably higher levels than normal skin, but COL-III is correspondingly lower in composition (*3*). As illustrated in fig. S18C, the elevated ratio of COL-I/III in wounds was effectively suppressed by MLVgel, indicating the tremendous potential for a scarless wound treatment effect of this platform.

In addition, hydrogels are regarded as an optimal choice for burn dressings due to their capacity to regulate water balance in the burned area, establish a moist microniche, facilitate rapid cooling, and alleviate persistent pain (*52*). To investigate the scar prevention efficacy of MLVgel on burn wounds, a deep grade II wound model was established on the back of Sprague Dawley (SD) rats and randomly divided into five groups. After treated with different formulations, it was noteworthy that all hydrogel treatment groups exhibited expedited wound closure and suppressed wound ulceration after 18 days of treatment, in which MLVgel exhibited the best outcomes contributed to the combined administration strategy and sequential release of MX and L-VP. On day 10, the skin in the control group still showed persistent necrotic epidermis, whereas new epithelial tissues were formed in the MLVgel-treated rats. Moreover, MLVgel effectively increased the number of new hair follicles and alleviated the abnormal proportion of COL-I/III, thus promoting burn wound closure and impeding scar formation (fig. S19).

Last, we also focused on the in vivo biosafety of MLVgel. Blood biochemical analysis indicated no significant differences between the MLVgel-involved group and healthy rats (fig. S20). Furthermore, H&E staining images of major organs (heart, liver, spleen, lung, and kidney) on day 14 revealed no significant changes in morphology and structure in the hydrogel-treated rats compared to the control group (fig. S21). These findings collectively suggested that our spatiotemporally released hydrogel exhibited an excellent biosafety profile.

### MLVgel restrained HS in rabbit ears

**Fig. 6.**
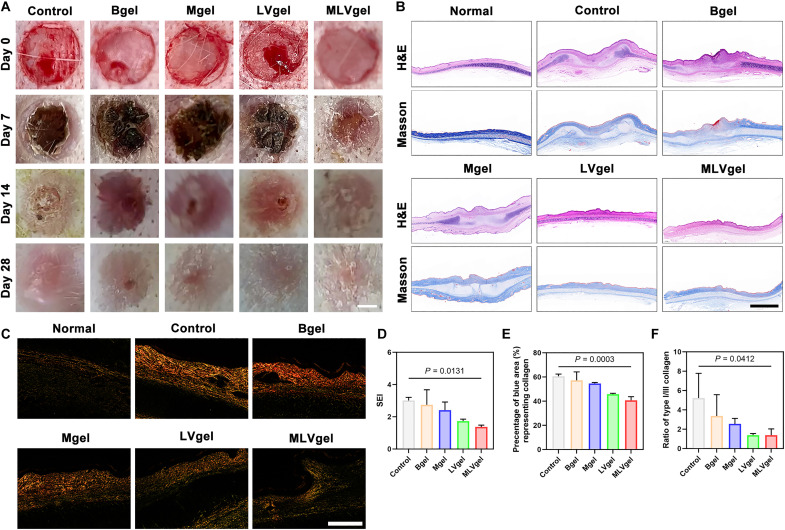
Hydrogel prevents HS formation in rabbit ear. (**A**) Reprehensive wound photographs of rabbit ear in various treatment groups at different time points. Scale bar, 2 mm. (**B**) Representative H&E and Masson tricolor staining images of wounds on day 28. Scale bar, 2 mm. (**C**) Representative Sirius Red staining images of wounds on day 28. Scale bar, 1 mm. Quantitative analysis of (**D**) scar hyperplasia index (SEI), (**E**) percentage of collagen, and (**F**) COLI-III ratio (*n =* 3). Data were presented as means ± SD, and statistical significance was analyzed via one-way ANOVA with Tukey’s multiple comparisons test.

Although rat wound models are commonly explored, the healing process of rat back skin is still quietly different from that of humans, attributed to the laxity of the back skin and the absence of mechanical tension (*53*). In comparison, the rabbit ear wound model is widely regarded as the most appropriate and convenient model for simulating HS in human. Here, tight and hairless skin on the inner side of the rabbit ear was used to fabricate the HS model, and the reepithelialization process was prolonged through the peeling of perichondrium. Subsequently, the rabbits were divided into five groups and treated with PBS, Bgel, Mgel, LVgel, and MLVgel, respectively. After the treatment of hydrogels, rabbits exhibited more desired healing behavior compared to the control group (Fig. 6A). On day 28, PBS-treated group displayed HS with a reddish hue, obvious protrusions, firm texture, and smooth touch, along with noticeable aggression toward adjacent normal tissue. Moreover, obvious HS red spots were still visible in the wound center after Bgel and Mgel treatment, which were invisible in the MLVgel group. The above conclusions indicated that gel components and doped MX could only promote wound healing but not restrain HS generation, suggesting that each component in the hydrogels was indispensable.

In contrast, the HS of MLVgel-treated rabbits was flatter and similar in color and tactility to normal tissue. H&E, Masson, and Sirius Red staining were used to assess the proliferative characteristics of different treatment groups. The PBS-treated group demonstrated a substantial increase in dermal thickness and a prominent wound bulge, both of which were markedly ameliorated by the application of MLVgel, as evidenced by scar hyperplasia index (Fig. 6, B and D). The Masson and Sirius Red staining results illustrated a reduction in collagen deposition in LVgel- and MLVgel-involved rabbits, with the more organized arrangement and more reasonable proportions of COL-I/III (Fig. 6, C, E, and F). We then disclosed the underlying mechanism for conquering HS. The decreased fibroblasts’ profibrotic gene expression [α-SMA, transforming growth factor–β (TGF-β), and COL-I] (*54*)] after the treatment of both LVgel and MLVgel detected by the immunofluorescence staining (fig. S22) reconfirmed the results of Sirius Red staining. Overall, our experiments proposed a classic model of the point for synergistic and spatiotemporal drug release, where the inflammation inhibition promoted wound reepithelialization, and long-term release of L-VP prevented the formation of HS.

**Fig. 7.**
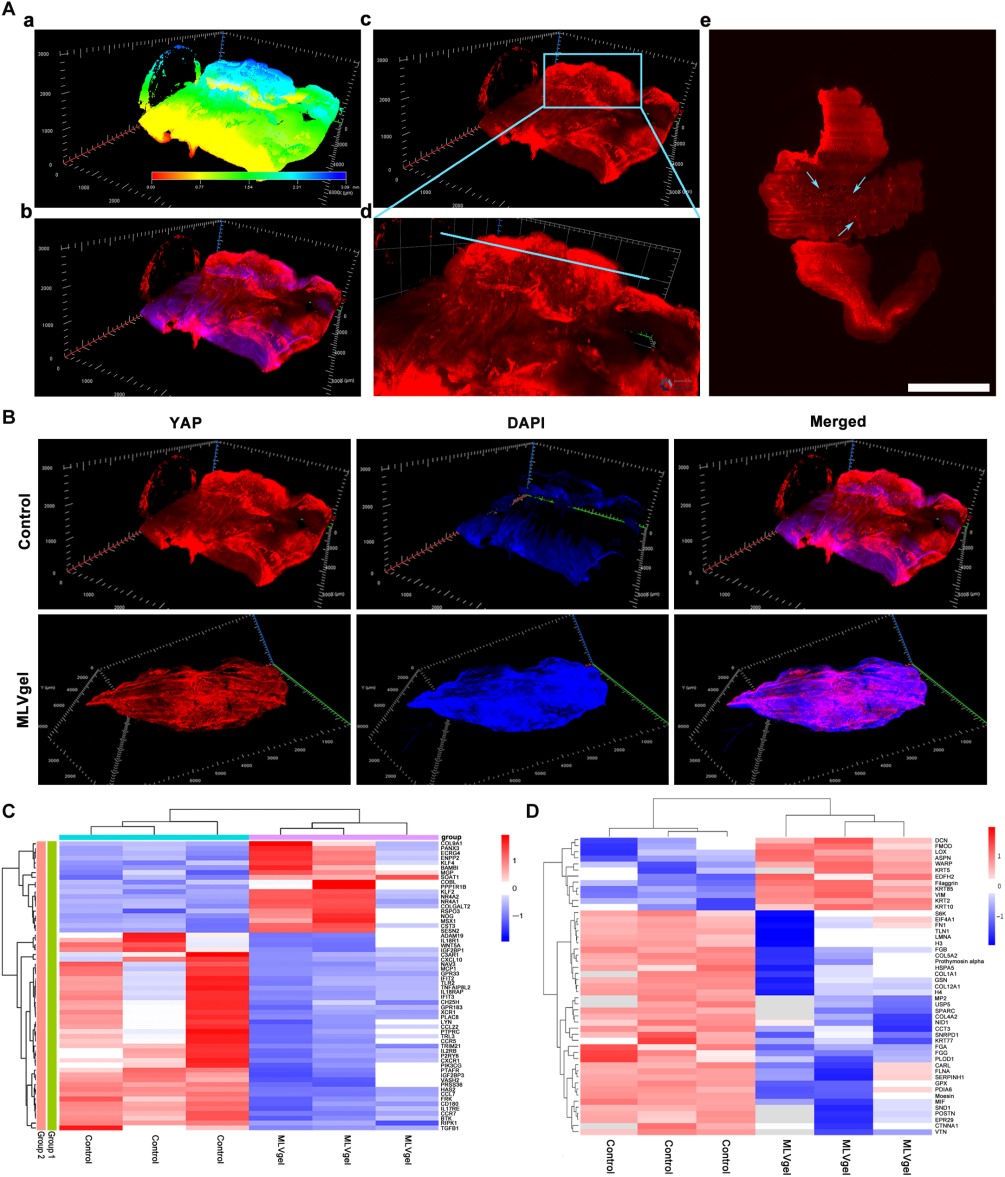
Biological protein expression of HS tissue in rabbit ears. (**A**) Representative flowchart of YAP-labeled HS imaging. (a) Tissue thickness map of HS tissues, where blue shift represents an increase in the *z*-axis scale. (b) Colocalized images of DAPI and YAP in HS tissues. (c) Distribution of YAP in HS. (d) Distribution of YAP in the scar after magnification of HS. The blue box in (A, c) represents the magnification range. (e) Cross-sectional image of YAP distribution in HS, and blue arrows mark vesicular structures with high YAP expression in HS. Scale bar, 1 mm. (**B**) Representative three-dimensional fluorescent staining images of YAP. (**C**) Heatmap analysis of differentially expressed genes (*n =* 3). (**D**) Heatmap analysis of differentially expressed proteins (*n =* 3).

A tissue clearing technique could reveal the spatial distribution of specific proteins in tissues. Here, we hyalinized rabbit ear scar tissues without cartilage (fig. S23) and marked YAP proteins. Notably, YAP expression in untreated scar tissues (Fig. 7A) demonstrated site-specific enrichment at regions of dermal elevation with a pronounced longitudinal orientation. Crucially, the cross-sectional images revealed a previously unreported discontinuous vesicle-like distribution pattern of YAP proteins. By contrast, MLVgel-treated tissues were flatter and had lower YAP expression (Fig. 7B).

Our work established that single inflammation modulation exerted negligible inhibitory efficacy on HS formation, whereas combined mechanotransducive regulation achieved superior prophylactic efficacy. To further elucidate the anti-HS mechanism of MLVgel, we further explored the inhibitory effect of MLVgel on scarring through transcriptomic and proteomic analyses, respectively. Ideally, the results revealed notable differences in gene and protein expression between PBS and MLVgel treatment groups. A transcriptomic volcano map illustrated 241 up-regulated genes and 298 down-regulated genes (fig. S24A). In addition, 155 proteins were down-regulated, while 46 proteins were up-regulated seen in the proteomic analysis (fig. S24B). Transcriptomic heatmaps revealed that in the MLVgel treatment group, TGF-β1 and immune-related genes including Toll-like receptor 3 (*TRL3*), *TRL4*, IL-17 receptor RE, *MCP-1*, chemokine 7 (*CCL7/MCP3*), interferon-induced protein with tetratricopeptide repeats 2 (*IFIT2*), *IFIT3*, and *CD180* were all down-regulated. It is noteworthy that the IL-17 family is not only associated with high inflammatory expression activity but also plays an important role in HS formation (*55*). Simultaneously, pannexin 3, esophageal cancer-associated gene 4, Kruppel-like factor, bone morphogenetic protein and activin membrane binding inhibitors, Msh homeobox 1, Sestrin2, and other genes related to immunoregulation and fibrosis inhibition were up-regulated (Fig. 7C). The differentially expressed genes were collected for analysis in the Gene Ontology (GO) database, which includes examination of biological processes, cell components, and molecular functions. We could conclude that genes associated with wound repair, such as the extracellular region and the developmental process, were shown to be up-regulated after treated with MLVgel. Conversely, terms related to an inflammatory-related immune process appeared to be down-regulated (fig. S24, C and D). Kyoto Encyclopedia of Genes and Genomes (KEGG) was chosen for the analysis of potential signal transduction pathways (fig. S24, E to G). The preventing effects of scar formation by MLVgel was intricately linked to reepithelization, involved in the endoplasmic reticulum, PI3K-Akt signaling pathway, nuclear factor κB signaling pathway, and TGF-β–related pathway.

Proteomic data provided us with another intriguing perspective (Fig. 7D). The family members of collagen, fibrinogen type B, heat shock 70-kDa protein 5, 1,2-oxyglutarate 5-dioxygenase 1, filamentin A, serine protease inhibitor peptidase inhibitor clade H, member 1, and other profibrotic proteins were down-regulated expression, accompanied by the up-regulation of core proteoglycan decorin (DCN), fibromodulin (*56*), cytokeratin family, filaggrin, and other proteins associated with antifibrosis and cuticle repair. In particular, the up-regulated DCN indicated the disappearance of HS, which may affect scar tissues by delaying the lateral assembly of collagen and blocking the TGF-β activity (*57*). These results all suggested that MLVgel may alleviate HS by reducing collagen deposition and abnormal tissue fibrosis. The GO database analysis of proteomics (fig. S24, H and I) suggested that the cluster of actin-based cell projections, fibrinogen complex, and actin-dependent adenosine triphosphatase activity was down-regulated in the MLVgel-involved group. Conversely, ECM structural constituent, tissue development, epithelium development, and skin development were up-regulated. It demonstrated that MLVgel held the potential to augment tissue regeneration, reepithelialization, and skin repair, accompanied by regulating the compositions of ECM, ultimately obstructing HS. Collectively, both proteomic and transcriptomic studies revealed an intriguing concept, indicating that HS wounds continued to display chronic inflammatory characteristics even during the remodeling phase, which is consistent with clinical reports of cicatricial itching (*2*). Furthermore, untreated HS tissues also exhibited increased expression of proinflammatory pathways and high levels of fibrosis and collagen deposition. While MLVgel rendered a promising modality for suppressing inflammation and fibrosis simultaneously, thus forwarding scarless tissue repair and reepithelialization with regeneration of secondary skin elements.

### Therapeutic efficacy of MLVgel on established scars in Bama miniature pigs

**Fig. 8.**
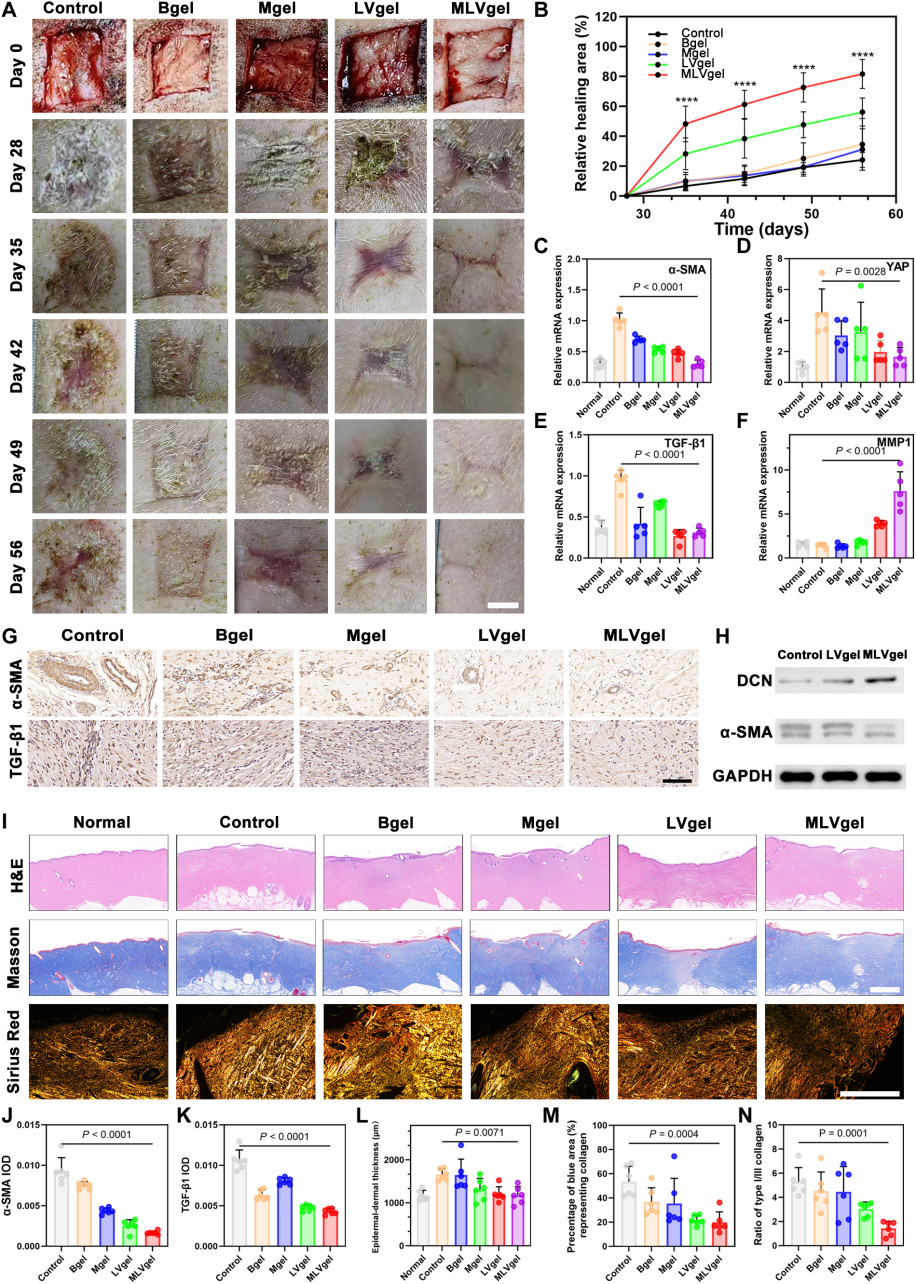
Hydrogel treatment of scarred pig skin. (**A**) Wound photographs of pig skin in various treatment groups at different time points. Scale bar, 1 cm. (**B**) Quantitative analysis of the relative wound healing rate at different times (*n* = 8). Relative mRNA expression of (**C**) α-SMA, (**D**) YAP, (**E**) TGF-β1, and (**F**) MMP-1 (*n* = 5). (**G**) Representative immunohistochemical staining images of wounds on day 56. Scale bar, 100 μm. (**H**) Western blot (WB) analysis of differential expression of α-SMA and DCN of wounds on day 56. (**I**) Representative H&E, Masson tricolor, and Sirius Red staining images of wounds on day 56. Scale bars, 2 mm. Integral optical density (IOD) analysis of (**J**) α-SMA and (**K**) TGF-β1 (*n* = 6). Quantitative analysis of (**L**) epidermal-dermal thickness, (**M**) percentage of collagen, and (**N**) COLI-III ratio (*n* = 6). Data are presented as means ± SD, and statistical significance was analyzed via one-way ANOVA with Tukey’s multiple comparisons test.

Given the abovementioned results, we have demonstrated the versatile efficacy of MLVgel in preventing scarring. However, the therapeutic potential against scar tissue remained to be fully elucidated, which may resolve the dilemma in clinical application. Here, the antiscar efficacy was evaluated in pig skins, which have similar physiology to human skin and comparable tension (*58*). Female Bama miniature pigs were naturally fed for 28 days after receiving medium and thick skin wounds on their backs. Then, they were randomly divided into five groups and treated with PBS, Bgel, Mgel, LVgel, and MLVgel, respectively. The wound healing area was measured at days 28, 35, 42, 49, and 56. Distinct differences were demonstrated in the wound closure among PBS-, LVgel-, and MLVgel-treated groups (Fig. 8, A and B). After 7 days of administration, LVgel- and MLVgel-treated pigs exhibited a notable relief of HS and an accelerated reepithelialization process owing to the targeted delivery of L-VP. Wounds treated with LVgel and MLVgel showed rapid healing at day 7 with some red scarring, which quickly subsided after 14 days of medication. However, the therapeutic efficacy of MLVgel was obviously better than that of LVgel ascribed to the adjuvant results of MX. After 28 days of treatment, no substantial therapeutic effect was observed in the control group, with a healing rate of less than 40%. In contrast, wounds in the MLVgel-involved group were nearly imperceptible (84.0 ± 8.6%), displaying a flat surface and no obvious disfigurement. As a consequence, it could deduce that the MLVgel manifested surpassing inhibitory effects on established scars.

The molecular mechanism was also initially investigated. Expressions of α-SMA, YAP, TGF-β1, and matrix metalloproteinase 1 (MMP1)–related genes in wound tissues were quantitatively analyzed by quantitative polymerase chain reaction (qPCR), where the MLVgel showcased the strongest regulatory capacity compared to other regimens (Fig. 8, C to F). The expression levels of YAP at the wound sites were down-regulated via the sustained release of VP, which may sequentially block *En1^+^* and expediting skin regeneration with less expressions of α-SMA and TGF-β. In contrast, MMP1, a gene associated with accelerated collagen and ECM production, was up-regulated. These findings were consistent with our previous proteomic analysis. Cross-validation was conducted through immunohistochemical staining and Western blotting (WB) assay, disclosing an obvious decrease in the expression of α-SMA, TGF-β, and DCN (Fig. 8, G to K, and fig. S25). Last, histological analyses were conducted using H&E, Masson, and Sirius Red staining. Similar to the prophylactic outcomes in HS on rabbit ears, H&E staining revealed hypertrophic fibroblast infiltration and epidermal and dermal hyperplasia in the PBS-treated group (Fig. 8I), with abnormal epidermal-dermal thickness (Fig. 8L). In contrast, MLVgel-treated pigs had a thinner and flatter dermis, along with reduced collagen deposition in wound sites (Fig. 8M) and decreased proportion of COL-I/III (Fig. 8N), deducing the notably therapeutic effect of the spatiotemporally released hydrogel.

## DISCUSSION

Skin wound healing is a highly intricate and programmed process consisting of four major stages, and it is crucial to recognize the vital role of niche homeostasis in the scarless wound healing behavior. Inflammation phase is compulsory for healing, and overexpressed proinflammatory factors during this phase and consequent uncontrolled proliferation of fibroblasts in the proliferative phase lead to the formation of HS (*2*). However, simultaneous studies targeting the regulation of immunity and fibrosis have rarely been reported. Thus, we integrated optimized anti-inflammatory modules in MLVgel which performed well against such conditions.

Bacterial infections and immune dysregulation are also important causes for wound healing stalls in the inflammatory phase, where conventional antibiotics sometimes fail due to drug resistance. In the case of MLVgel, the niche at the wound site breaks the boronic ester bonds to release COS and AMPs, which suppress bacteria growth together with the loaded MX (*25*). The selective inhibition of COX-2 can enhance the overall biosafety. Furthermore, we ensure that the screening of drug concentrations prevents excessive suppression of inflammatory responses. In addition, doping of hydrogenated soya phosphatidylcholine (HSPC)–contained liposomes creates a stable hydrated lubricating layer on the hydrogel surface (*44*), which helps to resist bacterial attachment and further prevent wound reinfection. MLVgel helps the regulation of the immune microenvironment and clear ROS at proper stage, to boost phase transition.

At the end stage, promoting recovery of functional hair follicles, reestablishment of normal matrix ultrastructure, and restoration of mechanical robustness are crucial for scar-free healing. YAP inhibition by VP could restrain *En1^+^* activation and *En1* lineage–negative fibroblasts-mediated repair (*59*). In another independent study, YAP was identified as a key factor regulating the transition of hair follicle placode from epithelial thickening to downward budding, thereby promoting cellular mitosis (*28*). Here, a three-dimensional distribution model of YAP constructed by tissue transparency reports that YAP is concentrated in the secondary structures of scars, which are called “vesicles” and are morphologically close to hair follicles. This discovery suggests that YAP may play a more critical role in HS formation, and its interaction with hair follicle stem cells regarding their influence on HS warrants further investigation. Meanwhile, the ROS produced by VP under laser irradiation is harmful to wound healing. We found that encapsulating VP into myofibroblast-targeted liposomes could turn off photosensitivity through the *homo*-FRET effect and maintain the YAP inhibition capacity (*16*). Notably, compared to fibroblasts, macrophages exhibited lower uptake capacity of positively charged L-VP than free VP. This phenomenon may result from the preferential binding of scavenger receptors to negatively charged nanoparticles, coupled with their high affinity for low-fluidity liposomal membranes (*34*, *36*). In addition, possible structure changes of VP may weaken or alter its inhibitory effect on YAP. These findings could be explored in future studies.

Last, in this study, the therapeutic effects of MLVgel were validated on the scar wound model in pigs which has greater similarity to human skins. This gives strong evidence for the potential of clinical translation of MLVgel. Further validation in primate models is feasible.

To sum up, we have developed an injectable spatiotemporal release hydrogel that can conform to irregularly shaped wounds and dynamically regulate the whole wound healing process. First, the MLVgel provides antimicrobial and immunomodulatory functions during the inflammatory period. Sequentially, controlled release of L-VP promotes scarless skin wound regeneration by inhibiting YAP signaling, with full recovery of secondary skin elements and mechanical strength. Simultaneously, the presence of hydrated lubrication layer inhibits the reinfection of wounds. The preventive and therapeutic efficiencies of MLVgel were verified in rat infected and burn wounds, HS prevention capacity in rabbit ears, as well as the HS treatment effect in Bama miniature pigs. Thus, this hydrogel is a promising method for scar prevention and repair in clinical practice and may be effective against keloid and psoriasis. Certainly, given the pivotal role of YAP in Hippo signaling transduction, its long-term clinical safety should be considered in the future. Nevertheless, the spatial distribution of YAP uncovered in this study laid a basis for investigating fibroblast activation signals. Furthermore, we also highlighted the importance of programed dynamic regulation in wound healing and the broad therapeutic potential of YAP/inflammation dual inhibition.

## METHODS

### Materials

YQT-12 (sequence: SLYQTDDRNDYIC) and KRL (sequence: KKLRLKIAFK) were commercially purchased by GL Biochem Co. Ltd., purified by high-performance liquid to more than 98%, characterized by high-performance liquid chromatography and mass spectroscopy supplied as a lyophilized powder. COS were purchased from Tokyo Chemical Industry Co. Ltd. Cholesterol, HSPC, DSPE-PEG2000, and DSPE-PEG2000-MAL were purchased from A.V.T. Medical Technology Co. Ltd. LB broth medium and LB agar plate medium were purchased from Huankai Microbial Technology Co. Ltd. All other reagents and solvents were purchased from Adamas and Chengdu Chron Chemical Co. Ltd.

### Synthesis of PBA-grafted KRL

The grafts were synthesized by the standard amidation reaction (*60*). Briefly, KRL (500 mg) and PBA (800 mg) were dissolved separately in dimethyl sulfoxide (DMSO; 50 ml), to which EDCI (924 mg) and NHS (554 mg) were added and the carboxyl group of PBA was activated by immersing the mixture into an ice bath for 1 hour. Then, KRL solution was added drop by drop to the system under nitrogen protection and stirred for 24 hours. After 3 days of dialysis, PBA-KRL was obtained with the method of lyophilization. The purity was characterized by 11B NMR (Bruker AV II-600 MHz), and chemical shifts were referenced to the external standard of BF3·Et2O.

### Synthesis of GA-grafted COS

Under nitrogen atmosphere, GA (210 mg) was dissolved in MES buffer [50 mM, (pH 5.5), 0.5 M NaCl, 50 ml] with EDCI (238 mg) and NHS (149 mg). After ice bath, the carboxyl group was activated for 1 hour, and COS (1170 mg) was further added to the aforesaid system and reacted for 24 hours (*61*). After 3 days of dialysis, GA-COS was obtained by freeze-drying and characterized by Fourier transform infrared spectroscopy (Bruker INVENIO R) and 1H NMR (Bruker AV II-600 MHz).

### Preparation of VP liposomes

l-VP was fabricated by thin-film hydration technology. HSPC, CHO, DSPE-PEG2000, and VP (weight ratio: 40:6:2:1) were dissolved in trichloromethane. After removing the organic solvent, the film is hydrated in deionized water and further ultrasonically broken into nanoparticles under an ultrasonic homogenizer (SCIENTZ-IID, 120 W, 12 min). The drug-free liposome, the DiD-labeled liposome, and the maleimide derivative liposomes were fabricated via the same method with HSPC, CHO, DSPE-PEG2000-MAL, and VP (weight ratio: 40:6:2:1). L-VP were prepared through the mercapto-maleimide click chemical reaction at room temperature for 4 hours. The particle size, potential, and poly-dispersion coefficient (PDI) of liposomes were measured by dynamic light scattering (Malvern Zetasizer Nano ZS90), and morphology was obtained by a transmission electron microscope (JEOL JEM-2100Plus).

For L-VP, free VP was removed from liposomes using Sephadex G-50 medium, and liquid chromatography followed by LC-MS (Thermo Fisher Scientific, TSQ02-10001) was used to quantify the encapsulation and loading efficiency. In the case 2,2,6,6-teramethly-4-pipridone was used as singlet oxygen catcher, dispersed VP was obtained by Triton X-100 demulsification of liposomes, and L-VP and empty liposomes were sufficiently irradiated by a 660-nm laser (PSU-III-LED) for 8 min, respectively. EPR spectroscopy (Bruker EMXplus X-band EPR) was used for identification of the singlet oxygen switching effect of liposomes. The fluorescence intensity of L-VP and discrete VP was also observed by small animal imaging (PerkinElmer IVIS Lumina III).

### Fabrication of the MLV hydrogels

A solution of GA-COS (10%, w/v) was prepared in distilled water (DW), and MX (1 mg ml^−1^) predissolved in DMSO was added to obtain GA-COS and MX solution. PBA-KRL predissolved in DMSO was diluted with DW (10%, w/v). Then, L-VP (20 μg ml^−1^) was further added, and the pH value was adjusted to 8 to obtain PBA-KRL and L-VP solution. The above two solutions are mixed in equal proportions to prepare MLVgel. Hydrogels without MX (LVgel), without L-VP (Mgel), or both (Bgel) were prepared in the same way.

### Characterization of hydrogels

To determine the stability of liposomes in hydrogels, hydrogel samples containing DiD-labeled liposomes were placed at 4°C for 30 days and then cut into 200-nm-thin slices using a vibratome (Leica VT1200S). The hydrogel morphology was observed using cryo-SEM (Thermo Fisher Scientific Apreo S HiVoc), and mapping was analyzed. The dispersion of liposomes was imaged by CLSM (Zeiss LSM800), and picture analysis was performed using Image Pro Plus6.0. Elemental analysis of lyophilized hydrogels was provided by XPS (Kratos AXIS Supra).

### In vitro of drug release

To evaluate the release and response behavior of hydrogels, the hydrogel was loaded into a dialysis bag and soaked in PBS (pH 7.4 or 6.5) with or without H_2_O_2_ (0.3 mM). The samples were placed on a shaking table at 37°C, and the media were collected at a predetermined time and supplemented with fresh media. After being demulsified with Triton X-100, the release curves for VP and MX were provided by LC-MS (Thermo Fisher Scientific, TSQ02-10001). Meanwhile, 250 μl of hydrogels was packed in a 1.5-ml centrifuge tube with PBS under different conditions and placed in a shaking table at 37°C for 24 hours. ROS (0.1, 0.3, 0.5, and 0.7 mM H_2_O_2_) and acid (pH 7.4, 6, 5.5, and 5) responses of the samples were observed.

### Rheological properties of the hydrogels

The modular intelligent advanced rotary rheometer (Anton Paar MCR302) was used to measure the energy storage and energy consumption modulus of the hydrogel at 25°C. The frequency sweep was measured with a scan frequency from 0.1 to 100 rad s^−1^ and a strain of 1%. Moreover, a continuous step strain experiment was performed to determine the self-healing ability by circulating the small strain (1%, 60 s) and the large strain (300%, 60 s). The gelation time was measured at a scan frequency and a strain of 1%.

### Adhesion and wear resistance of hydrogels

The bioadhesion of the hydrogel was tested using female SD rat tissue. The coefficient of wet friction with or without liposome hydrogel was measured by universal friction and a wear tester (Bruker CETR UMT). Briefly, after the hydrogel is cured in the liquid tank (length: 30 mm, width: 30 mm, height: 4 mm), the friction force *F* between the sample and polished stainless-steel ball (304, diameter: 8 mm) under constant load (*F_n_* = 0.1 N) is measured at a sliding speed (1 mm s^−1^, path: 10 mm, cycle). The contact area *V_c_*, indentation depth *h*, and friction coefficient μ are calculated according to the formulas provided in the literature (*43*).

### Swelling and degradation behavior

After weighing the original weight, lyophilized bulk hydrogel was placed on PBS (pH 7.4) at 37°C. The swelling hydrogel was periodically removed, and the surface moisture was wiped off, recording immediate weight. As for degradation experiments, hydrogels were put into PBS (pH 7.4) at 37°C, took out, and lyophilized at specific times and weight.

### Hemolysis test

RBCs were obtained by rat blood centrifugation (1000 rpm, 5 min) and washed twice with PBS. The concentration of RBCs was adjusted with PBS to 5%. The lyophilized hydrogels (5 mg) was dispersed in PBS (500 μl) and evenly mixed with the RBC suspension in equal proportions. DW was used as positive control and PBS as negative control. After incubation in a shaking bed at 37°C for 4 hours, RBCs were centrifuged (1000 rpm, 10 min), and the RBC status was observed. The supernatant was transferred to a 96-well plate, and an absorbance of 540 nm was read by an enzyme labeler.

### Preparation for cell experiments

In this study, most situations, L929 (cl-0137, Procell) and RAW264.7 (cl-0190, Procell), were coincubated with hydrogel extracts as reported in the literature (*62*). In short, the sterilized hydrogel was soaked in Dulbecco’s modified Eagle’s medium (DMEM) (PM150210, Procell) culture (0.2 g ml^−1^) with or without serum and extracted for 24 hours.

### Cellular uptake assay

To verify the targeting of liposomes and free VP, modified and unmodified liposomes of targeted peptides were dispersed in DMEM culture. Precultured L929 cells were incubated for 24 hours and 30 min. After that, the medium was removed and rehung with sterile PBS (pH 7.4). Cell data were obtained by flow cytometry (BD FACS Celesta) and analyzed by FlowJo10. Fluorescence signals of cells were observed by CLSM (Zeiss LSM800) and quantitatively analyzed by ImageJ. The same flow assay also was performed on RAW264.7 cells.

### Cytotoxicity assay

The cytotoxicity of hydrogel was determined by a cell counting kit-8 (CCK-8) method. A total of 10^4^ L929 cells were inoculated into 24-well plates, cultured at 37°C for 24 hours, and further leaked into the extraction medium of hydrogels of different components for 3 days. The cells were washed with PBS and then incubated in DMEM complete medium containing 10% CCK-8 for 45 min, and the absorbance of 450 nm was read by an enzyme labeler. The cytotoxicity of different concentrations of the drugs was also tested under the same experimental condition.

### Evaluation of antioxidant activity

Liquid nitrogen quick-frozen hydrogel after homogenization (50 mg) was incubated with DPPH (100 μmol) in ethanol solution (2 ml) for 30 min. With vitamin C as positive control, the absorbance of 515 nm was read by an enzyme labeler. Antioxidant capacity was also indicated by DCFH-DA as the ROS indicator. L929 cells were cultured in confocal dishes for 24 hours. Next, H_2_O_2_ solution (0.3 mM) was added to the cell culture medium to activate the cells. After 30 min, the medium containing H_2_O_2_ was replaced with a liquid medium containing homogenate hydrogels (30 mg ml^−1^), and incubated for another 30 min. Last, the cell medium was replaced with the cell medium containing DCFH-DA (0.1 mM) solution and incubated for 30 min. The green fluorescence signal of cells was observed by an inverted fluorescence microscope (Nikon Ti-U). Fluorescence intensity was analyzed by ImageJ.

### Immunoblot analysis

L929 cells were incubated in six-well plates for 24 hours. Then, different concentrations of VP were added for another 24 hours. The protein was extracted at 4°C for 20 min by high-efficiency radioimmunoprecipitation assay lysis buffer (150 μl). The total protein content was determined by BCA protein assay. The Simple Western (ProteinSimple Western) was used for capillary immunoassay with an anti-rabbit detection module, and the protein expression was determined by a chemiluminescence method. The assay was with the following primary antibodies: glyceraldehyde phosphate dehydrogenase (GAPDH; 5174, Cell Signaling Technology), TAZ (72804, Cell Signaling Technology), α-SMA (19245, Cell Signaling Technology), and SMAD2/3 (8685, Cell Signaling Technology).

### In vitro immunofluorescence assay

Equal concentrations of VP (5 μg ml^−1^) were fully irradiated with or without a laser (660 nm) for 24 hours, and L929 cells were incubated for 24 hours and washed three times with PBS. The expression of YAP (GB11542-50, Servicebio) was detected by immunofluorescence staining. The images were recorded using a digital microscanner (PANNORAMIC MIDI II, 3D-HISTECH).

### In vitro immune regulation of hydrogels

Mouse-derived macrophages (RAW264.7) was cultured in 24-well plates and incubated at 37°C for 24 hours. Then, the cells were pretreated with DMEM containing LPS (10 μg ml^−1^) for 2 hours. After washing with PBS, cells were then cultured with a medium containing a hydrogel extract for 24 hours. Last, the cell supernatant was collected, and the content of target protein was determined by ELISA kits. The cells were suspended with flow buffer (2% fetal bovine serum in PBS), stained, and further analyzed by flow cytometry (BD FACS Celesta). Meanwhile, real-time qPCR assay also followed the above steps to incubate cells. Total RNA was extracted using TRIzol reagent. The mRNA expression data were provided using a fluorescent quantitative PCR instrument (Roche LightCycler 96).

Staining was performed with the following flow antibodies: F4/80 (566787, BD Biosciences), CD86 (740034, BD Biosciences), and CD16/CD32 (553141, BD Biosciences). The following PCR primer sequences were used, with each gene being forward and reverse sequences, respectively:

*MCP-1* mouse, TTAAAAACCTGGATCGGAACCAA and GCATTAGCTTCAGATTTACGGGT; *IL-6* mouse, TAGTCCTTCCTACCCCAATTTCC and TTGGTCCTTAGCCACTCCTTC; *TNF*-α mouse, ACCCTCACACTCACAAACCCAC and ACAAGGTACAACCCATCGGC; *GAPDH* mouse, AGGTCGGTGTGAACGGATTTG and GGGGTCGTTGATGGCAACA.

### In vitro antibacterial activity assays

The bacteria were resuscitated and incubated in LB broth at 37°C until logarithmic growth (optical density at 600 nm = 0.8). Then, the bacterial concentration was adjusted to 10^8^ colony-forming units (CFU)/ml, and the pH value was adjusted to 6. Coincubated with hydrogel with or without the drugs, vancomycin and methicillin were considered as positive controls. After 12 hours, the bacterial suspension was collected, and the absorbance at 600 nm was read by the enzymograph. Furthermore, another bacterial suspension was coated on an LB agar plate and incubated for 12 hours until visual colonies were formed, and the LB agar plate was photographed. The experiment was performed with the following bacteria: Gram-positive *S. aureus* [American Type Culture Collection (ATCC) 25923], Gram-negative *P. aeruginosa* (ATCC 27853), and *MRSA* (ATCC 43300).

### Infected wound healing model

With the approval of the Animal Ethics Committee of Sichuan University (KS2023329), SD rats (female, 3 to 4 months, 200 to 220 g) were used in the experiment. The rats were randomly divided into six groups (*n* = 8) and anesthetized with pentobarbital (3%, 30 mg kg^−1^). After hair removal, two full-layer skin defects (diameter = 10 mm) were created on the back of the rats and inoculated with *P. aeruginosa* (100 μl, 10^8^ CFU ml^−1^). After 24 hours, PBS, Bgel, Mgel, LVgel, and MLVgel were respectively given. The wound area was photographed and measured on days 3, 7, and 14. Meanwhile, wounds of different treatment groups at different times were collected and stored in 4% paraformaldehyde (PFA) for H&E staining, Masson tri-color staining, and Sirius Red staining. A polarized light microscope (DM2700P, Leica) was used to capture images of Sirius Red staining, and other histological sections were recorded with a digital microtome scanner (PANNORAMIC MIDI II, 3D-HISTECH) and analyzed with ImageJ.

### In vivo anti-inflammatory and antibacterial activity

Under sterile conditions, tissues were collected after 3 days of different treatments and homogenized with PBS (500 μl). Part of the homogenate was diluted to a specific concentration, incubated on LB agar plates for 12 hours, and photographed. ImageJ was used for counting analysis. The remaining homogenate was used for quantitative analysis of inflammatory factors, which were detected by ELISA kits (MCP-1, TNF-α, IL-6, and IL-10). Meanwhile, the wound tissue was collected and chopped on day 3 and digested with type IV collagenase/deoxyribonuclidenase I (5 ml) for 45 min. The flow buffer was terminated, and a single-cell suspension was obtained after filtration with a 70-μm filter. All data were obtained by flow cytometry (BD FACS Celesta) and analyzed by FlowJo10. Otherwise, immunofluorescence staining about TNF-α (3707, Cell Signaling Technology), CD86 (ab220188, Abcam), CD206 (24595, Cell Signaling Technology), CD31 (11585, Cell Signaling Technology), and α-SMA of the wound tissue on day 7 was also used to analyze the anti-inflammatory effect of the hydrogel. Sections were recorded with a digital microtome scanner (PANNORAMIC MIDI II, 3D-HISTECH) and analyzed with ImageJ.

### In vivo biosafety evaluation

To assess the biosafety behavior of hydrogels, the blood of rats after 3 days of different treatments was collected and analyzed. Meanwhile, major organs (heart, liver, spleen, lung, and kidney) of rats on day 14 were collected for H&E staining. All histological sections were recorded with a digital microtome scanner (PANNORAMIC MIDI II, 3D-HISTECH).

### Hydrogel accelerates burn healing

SD rats (female, 3 to 4 months, 200 to 220 g) were used in the experiment. The rats were randomly divided into six groups (*n* = 6) and anesthetized with pentobarbital (3%, 30 mg kg^−1^). After hair removal, a constant temperature electric soldering iron [ZSER, 220 V temperature = 98°C, diameter of soldering iron (10 mm)] was applied flat to the back for 8 s to create deep II burn wounds. Immediately, PBS, Bgel, Mgel, LVgel, and MLVgel were administered separately. Wound was photographed and measured on days 5, 10, and 18. At the same time, wounds of different treatment groups at different times were collected, preserved with 4% PFA, and stained with H&E, Masson tri-color, and Sirius Red. All tissue sections were recorded with a digital microscanner (PANNORAMIC MIDI II, 3D-HISTECH) and analyzed with ImageJ.

### Reduce scarring with hydrogel prevention

With the approval of the Animal Ethics Committee of Sichuan University (Gwll2023076), New Zealand large-eared rabbits (female, 2 to 3 months, 2 to 2.5 kg) were randomly grouped and anesthetized with pentobarbital (3%, 30 mg kg^−1^), each ear was pierced (*n* = 6) with a trephine, and a periosteal stripper was used to remove the perichondrium (*63*). After hemostasis, PBS, Bgel, Mgel, LVgel, and MLVgel were administered respectively. Wound was photographed and measured on days 0, 7, 14, and 28. On day 28, the wounds were collected and preserved with 4% PFA and stained with H&E, Masson tri-color, Sirius Red, and immunofluorescence TGF-β (81746-2-RR, Proteintech), α-SMA, and COL-I (67288-1-Ig, Proteintech). All tissue sections were recorded with a digital microscanner (PANNORAMIC MIDI II, 3D-HISTECH) and analyzed with ImageJ.

### Transparency in organization

Rabbit ear scar tissue was collected, and the cartilage was carefully peeled at day 28. After PFA fixation, permeabilization, antibody labeling, and staining were performed. Hyaline tissue clearing solution (Hyaline Biotechnology Co.) was used to transparent HS tissue. Tissue was imaged by a digital light sheet laser fiber imaging system (Light sheet 7, Zeiss) and analyzed with the Zen system.

### Proteomics and transcriptomics

Rabbit ear scar tissue was collected at day 28 and washed with saline (4°C). Before sequencing, tissues were quick-frozen by liquid nitrogen and stored at −80°C (MONITOR HELIX, Shanghai). Transcriptome data analysis differential expression analysis was conducted using R Bioconductor package “edgeR” or “DESeq2.” The R package “ClusterProfiler” was applied for GO enrichment, topGO enrichment, and KEGG pathway enrichment analysis.

### The therapeutic effect of hydrogel on HS

To further explore the potential of hydrogels, with the approval of the Laboratory Animal Use and Management Committee of Chengdu Senwei Laboratory Animal Co. (IACUC-SWLAB-20240424001), Bama miniature pigs (female, 4 months, 13 to 15 kg) were randomly grouped, placed on a surgical stent, and anesthetized with pentobarbital (3%, 30 mg kg^−1^) (*58*). After the back skin was prepared, the medium and thick skin (length: 20 mm, width: 20 mm) were taken with a surgical scalpel, and the wound spacing was 20 mm. A total of eight independent wounds were taken from each pig. After 28 days of natural recovery, the wound healed and formed a HS. PBS, Bgel, Mgel, LVgel, and MLVgel were administered respectively. The hydrogel was fixed with oil gauze and bandage, and the same procedure was applied to the control group. The wounds were photographed and observed on days 35, 42, 49, and 56. On day 56, the wounds were collected, preserved with 4% PFA, and stained with H&E, Masson tri-color, Sirius Red, and immunohistochemistry (TGF-β and α-SMA). All tissue sections were recorded with a digital microscanner (PANNORAMIC MIDI II, 3D-HISTECH) and analyzed with ImageJ. In addition, 56 day wound tissues were collected and frozen at −80°C for qPCR and WB analysis.

The following PCR primer sequences were used, with each gene being forward and reverse sequences, respectively: *TGF-*β*1* pig, GCCTGAGGCCGTACTGGCTCTTT and GTGGGGGGTGCCCTTGAATTTAT; α*-SMA* pig, GGTTCTGGGCTCTGTAAGGC and GTCACCCACGTAGCTGTCTT; *MMP-1* pig, TGCTTCTGCTGCTGCTGCTTCTC and TCCACTGGCACCCCATCACTATT;

*YAP pig*, CAGGATGGCGGGACTCAAAA and CTGCTCATGCTTAGTCCGCT; *GAPDH pig*, TCTGCCGATGCCCCCATGTTTGT and ACAGTCTTCTGGGTGGCAGTGAT.

### Statistical analysis

Statistical analysis was performed using GraphPad Prism software, Origen software, and ImageJ software. All data were from at least three independent biological replicate experiments and expressed as means ± SD. Two groups were compared using the *t* test. Multiple comparisons were performed using one-way, bilateral analysis of variance (ANOVA) and Tukey’s multiple comparisons test. The probability value *P* < 0.05 was considered statistically significant.
