# Supplementary material for: Mechanoregulative hydrogel facilitates rapid scarless healing by self-adaptive control of wound niche at different stages
Source: Sci Adv. 2025 May 23;11(21):eadv9895. doi: 10.1126/sciadv.adv9895 (PMC13109952; doi:10.1126/sciadv.adv9895)
Supplement: Supplementary file 1 — Figs. S1 to S25 [file sciadv.adv9895_sm.pdf]

Science Advances
AAAS

Supplementary Materials for

Mechanoregulative hydrogel facilitates rapid scarless healing by self-adaptive control of wound niche at different stages

Haozhou Shu *et al.*

Corresponding author: Shiqi Huang, huangshiqi@scu.edu.cn; Ling Zhang, zhangling83@scu.edu.cn

Sci. Adv. **11**, eadv9895 (2025)
DOI: 10.1126/sciadv.adv9895

This PDF file includes:

Figs. S1 to S25

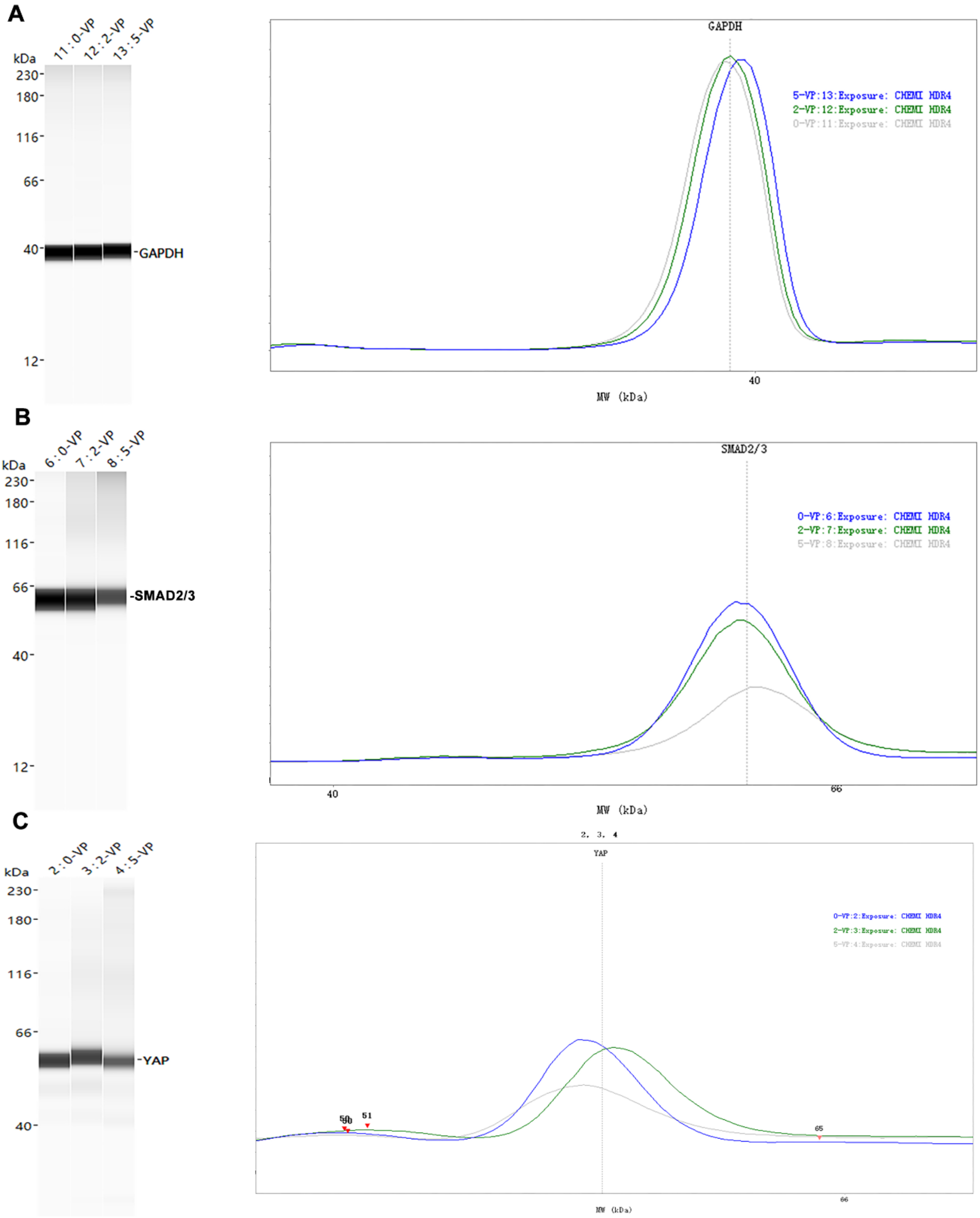
A
11 : 0-VP
12 : 2-VP
13 : 5-VP
kDa
230
180
116
66
40
12
GAPDH
GAPDH
5-VP:13:Exposure: CHEMI HDR4
2-VP:12:Exposure: CHEMI HDR4
0-VP:11:Exposure: CHEMI HDR4
40
MW (kDa)
B
6 : 0-VP
7 : 2-VP
8 : 5-VP
kDa
230
180
116
66
40
12
SMAD2/3
SMAD2/3
0-VP:6:Exposure: CHEMI HDR4
2-VP:7:Exposure: CHEMI HDR4
5-VP:8:Exposure: CHEMI HDR4
40
66
MW (kDa)
C
2 : 0-VP
3 : 2-VP
4 : 5-VP
kDa
230
180
116
66
40
YAP
2, 3, 4
YAP
51
65
66

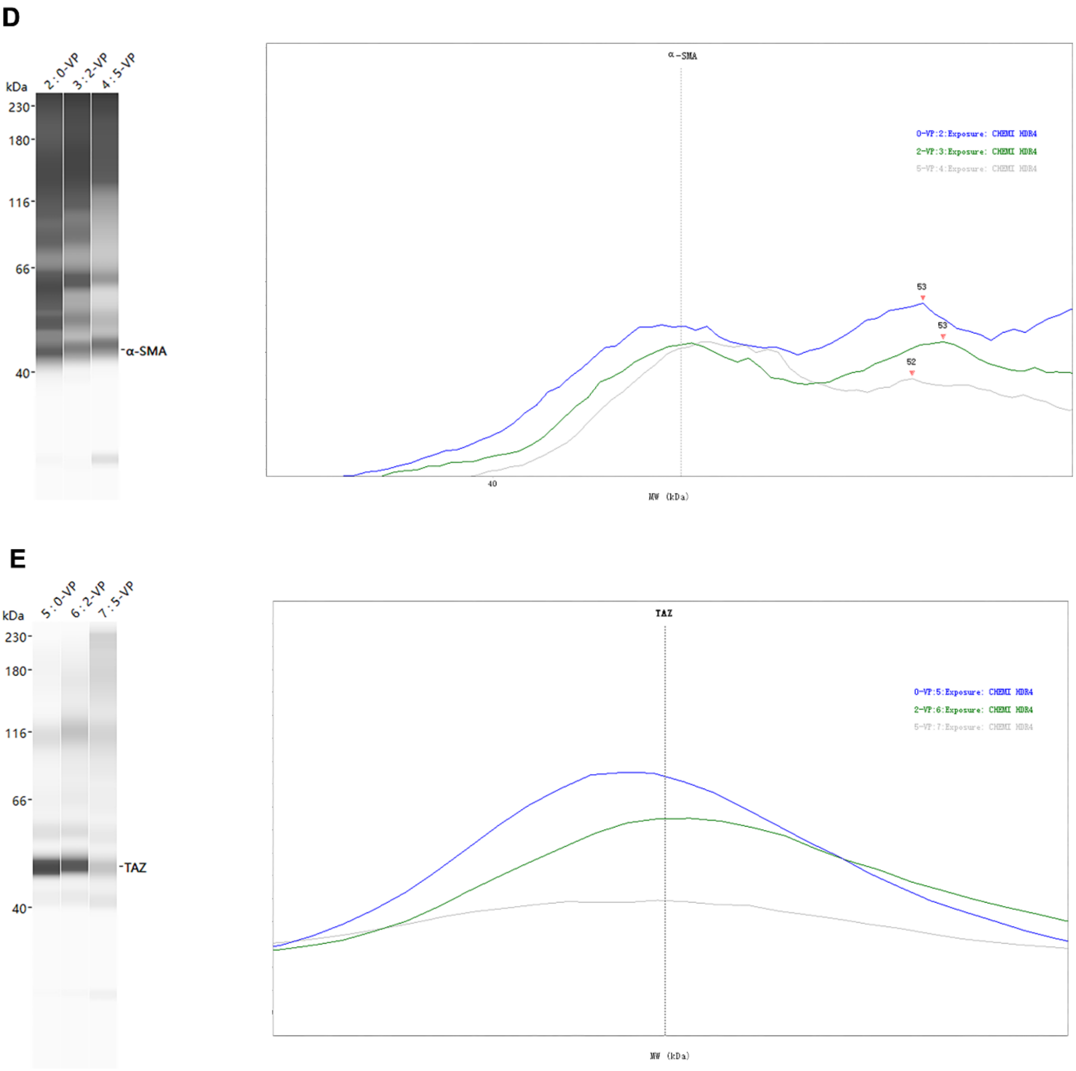

fig. S1. SimpleWesten results for full membrane and integral area of relevant proteins. A) GAPDH, B) SMAD2/3, C) YAP, D) α-SMA, E) TAZ.

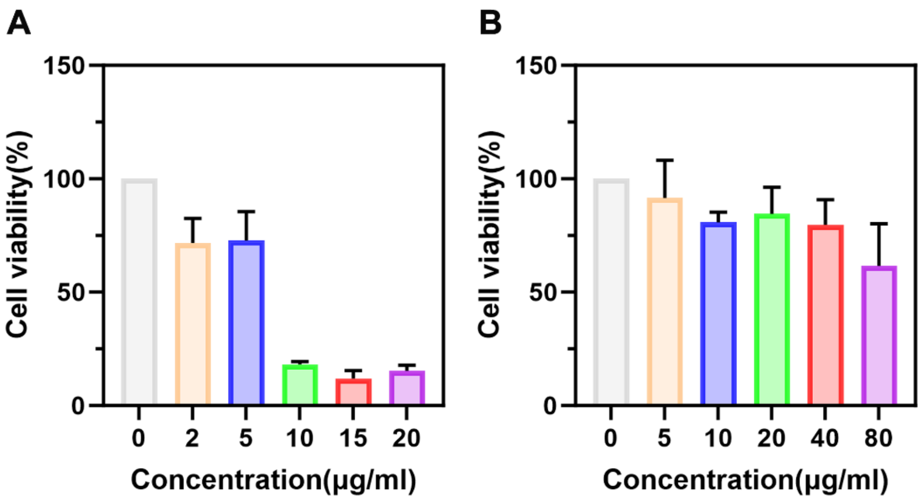

fig. S2. In vitro cytotoxicity assay. Cytotoxicity of A) VP and B) MX at different

concentrations to L929 fibroblasts ($n = 4$).

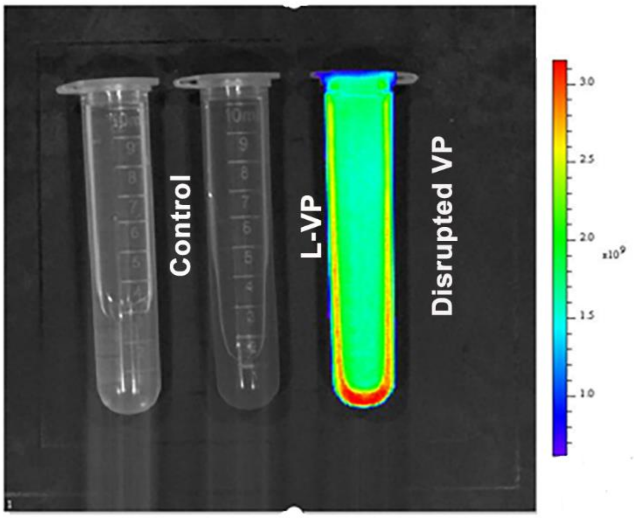

fig. S3. Fluorescence shielding of L-VP. Fluorescence image of blank liposomes, intact and disrupted L-VP.

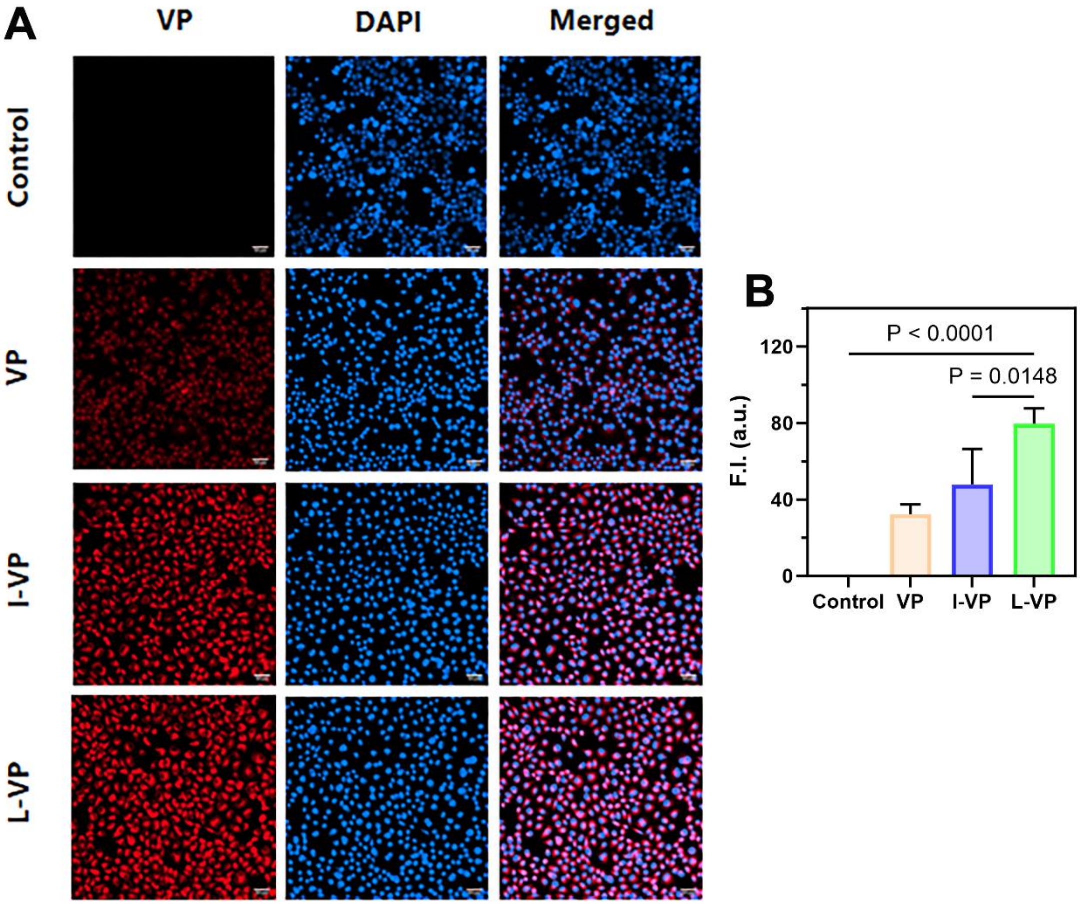

fig. S4. Cellular uptake of L-VP. A) Representative CLSM images of L929 cell

uptake. Scale bar, 50μm. B) Statistical analysis (n = 3). Data were presented as mean ± SD and statistical significance was analyzed via one-way ANOVA with Tukey's multiple comparison test.

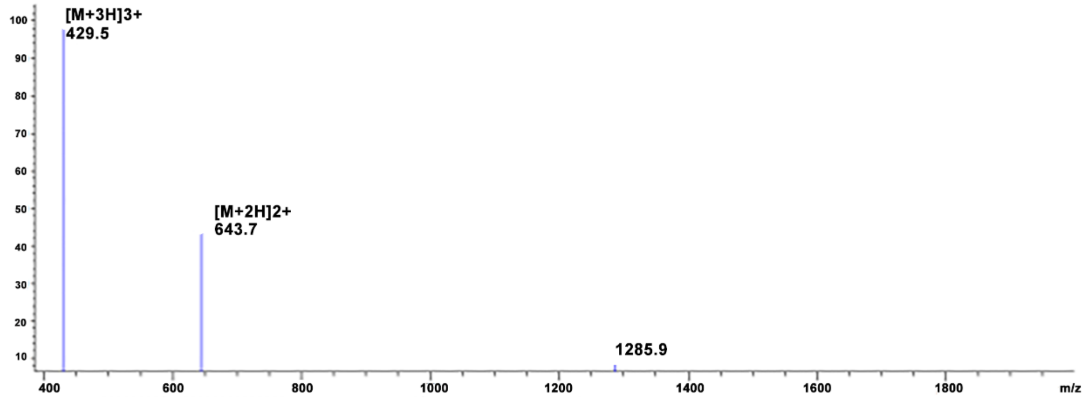

fig. S5. MS spectra of AMPs.

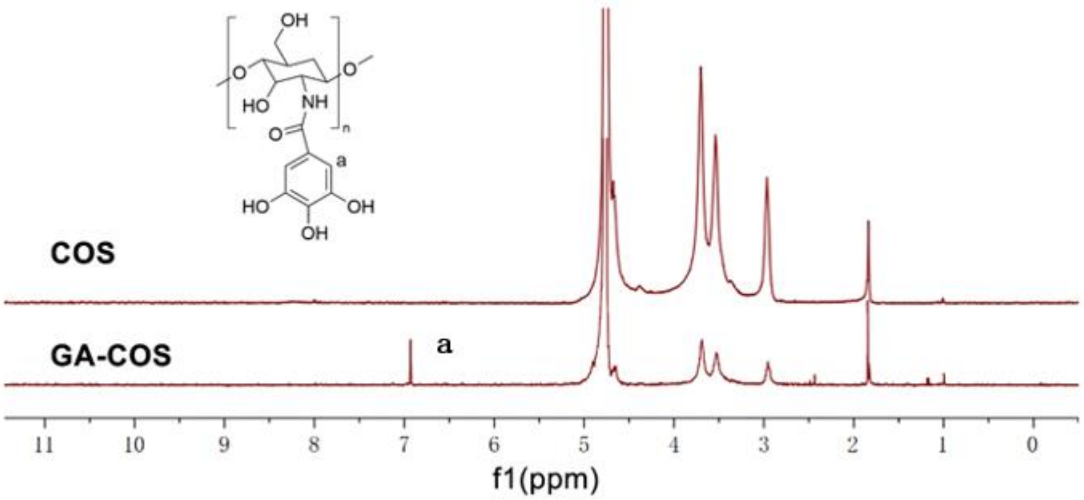

fig. S6. ^{1}H NMR spectra of COS and GA-COS.

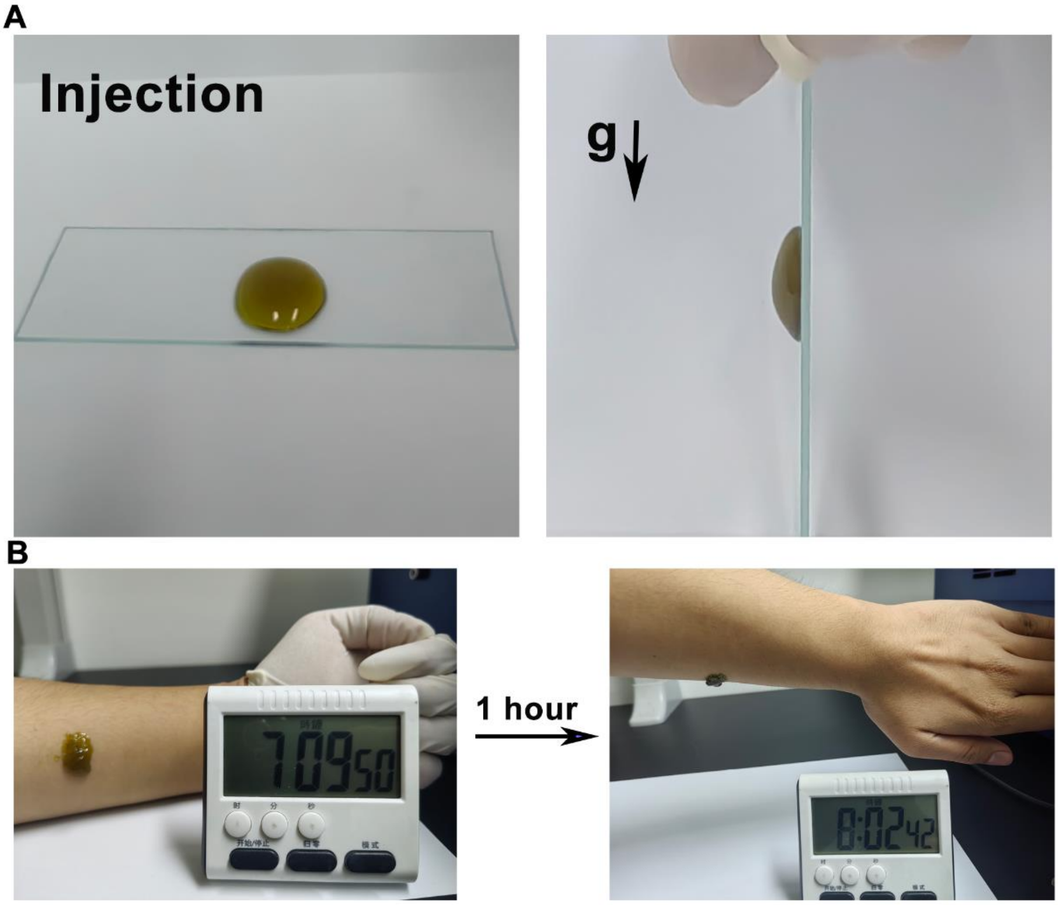

fig. S7. Adhesion of MLVgel. A) Picture of the material adhesion of hydrogels. B) Picture of human skin adhesion.

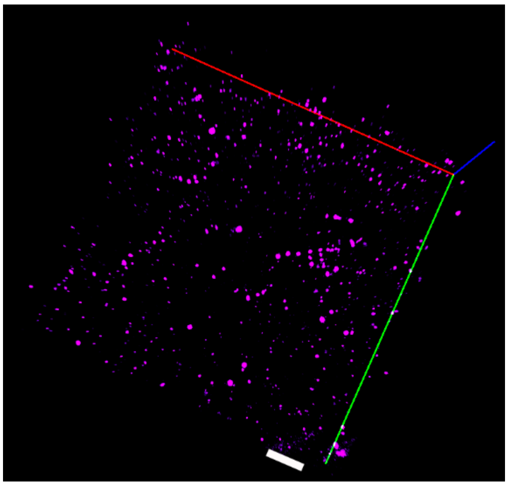

fig. S8. Long-term stability of L-VP in hydrogels. 3D confocal microscopy images of L-DiD@gel. Scale bar, 1μm.

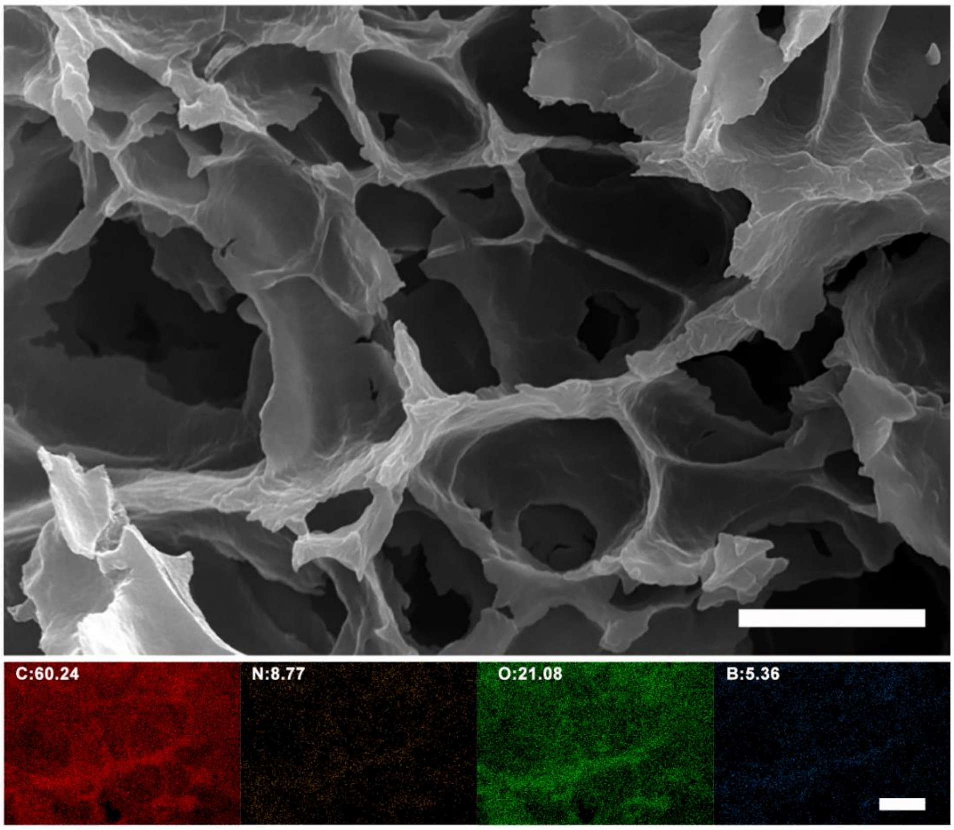

fig. S9. Elemental analysis of MLVgel. Representative SEM images and elemental analysis of freeze-dried hydrogels. Scale bar, 5μm.

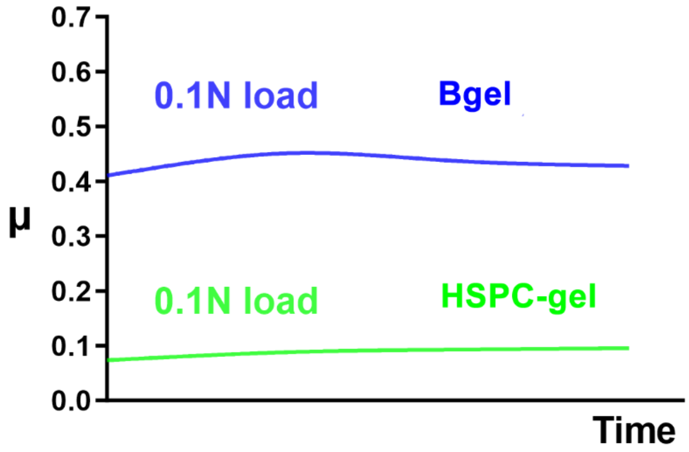

fig. S10. Hydration lubrication of hydrogels containing liposomes. Typical friction-time trajectories of hydrogels with or without liposomes content. Reciprocating step h:1cm. Sliding speed v_s:0.1cm/s.

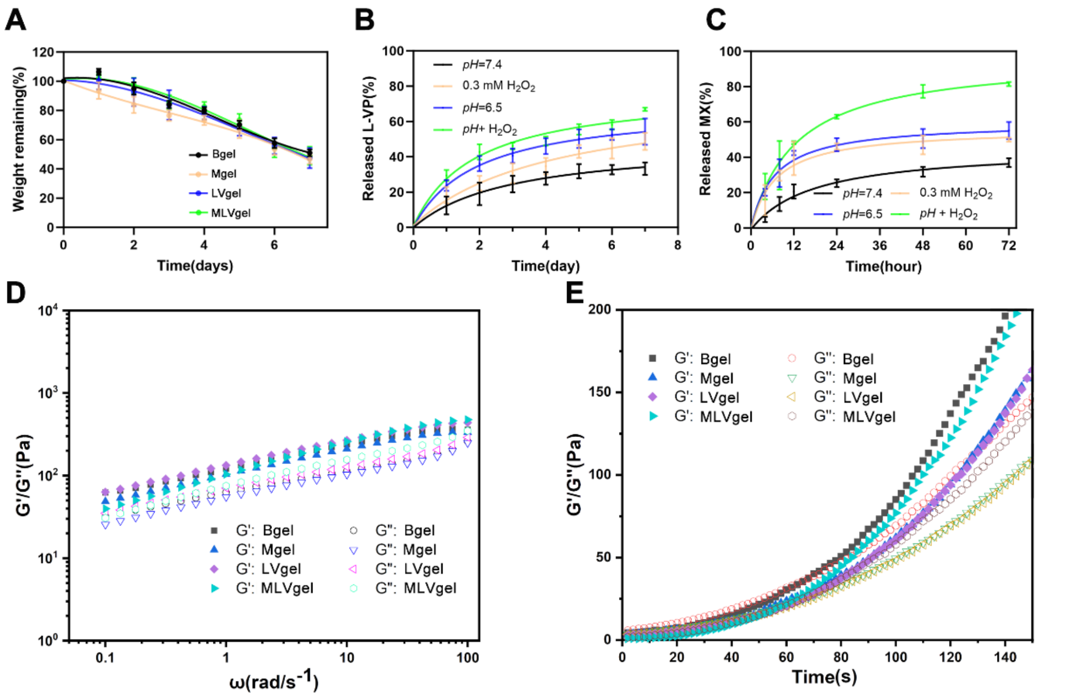

fig. S11. Characterization of the physical properties of hydrogels *in vitro*. A) Degradation rate of different hydrogels in physiological condition (PBS, pH=7.4) (n = 3). Release behavior of B) L-VP and C) MX under different conditions(n=3). D) Hydrogel frequency scanning experiments, ranging from 100 to 0.1 rad s^{-1}. E) Hydrogel formation time determination. T_m=25℃. Strain:1%, ω=1 rad s^{-1}. Data were presented as mean ± SD and fitted by non-linear regression.

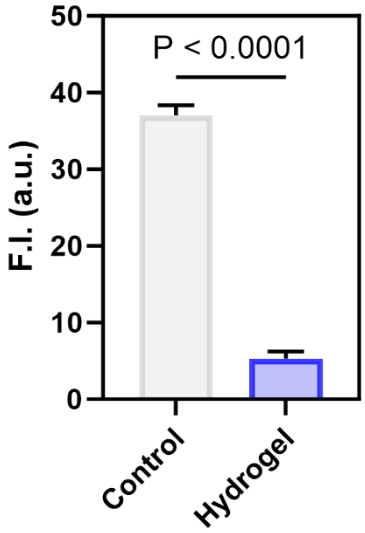

fig. S12. Antioxidant properties of hydrogels. Quantitative statistical analysis of DCFH-DA fluorescence with or without hydrogel treatment (n = 3). Data were presented as mean ± SD and statistical significance was analyzed via t test.

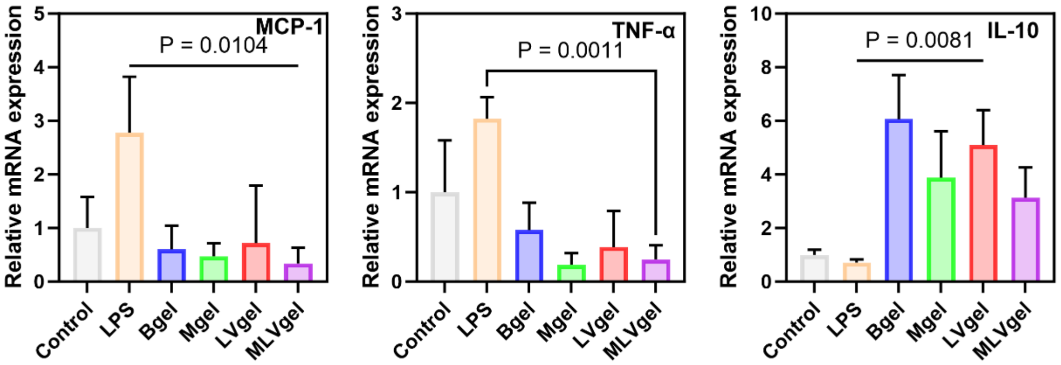

fig. S13. Expression of inflammation-related mRNA. Relative mRNA expression of MCP-1, TNF-α and IL-10 in RAW264.7 macrophages (n = 3). Data were presented as mean ± SD and statistical significance was analyzed via one-way ANOVA with Tukey's multiple comparison test.

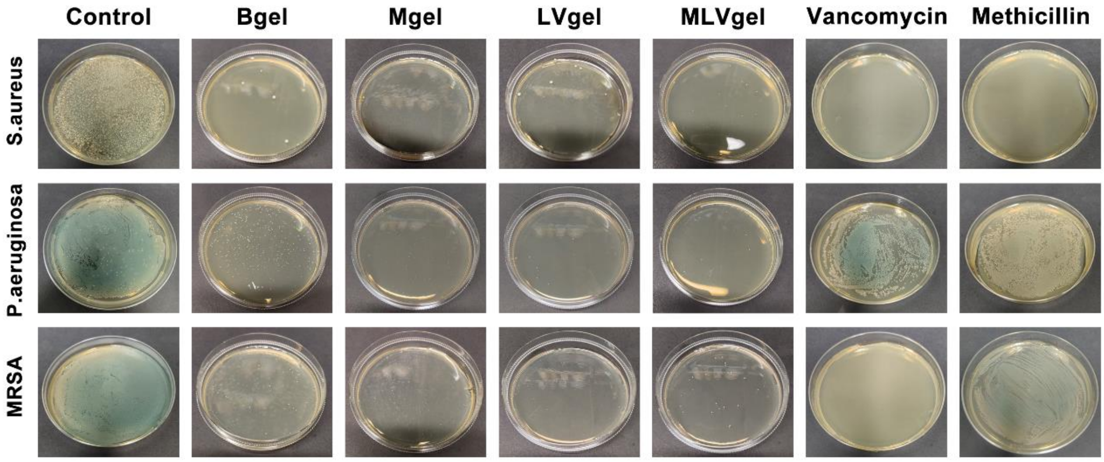

fig. S14. In vitro antimicrobial testing of hydrogels. Images of *S. aureus*， *P. aeruginosa*, *MRSA* on LB agar plates after incubating with the hydrogels or antibiotics for 24 h.

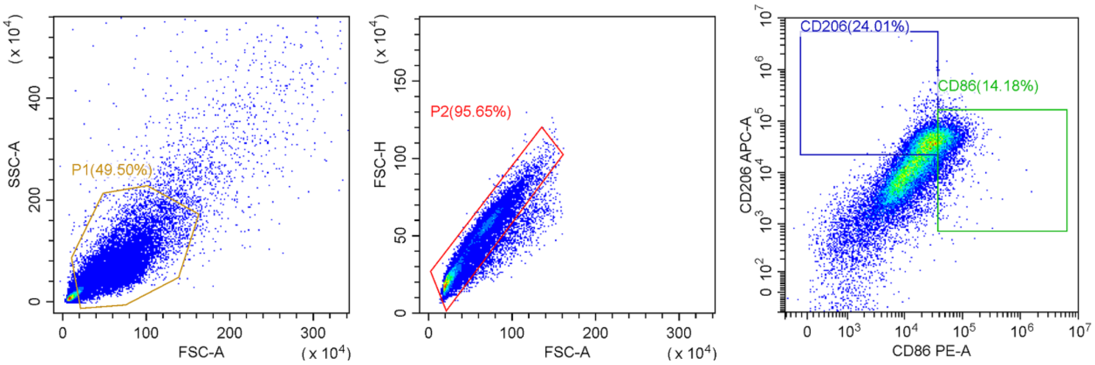

fig. S15. Gating strategies for macrophage typing in infected wound models of SD rats.

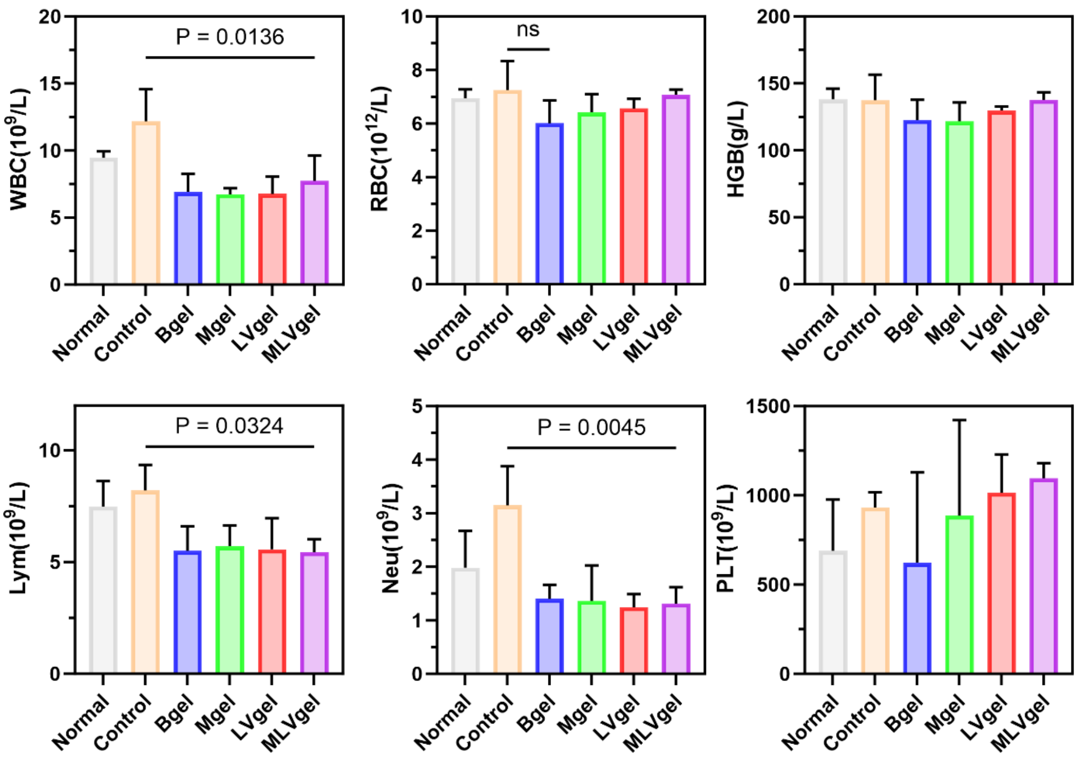

fig. S16. ***In vivo* blood routine analysis.** Blood routine (WBC: white blood cell, RBC: red blood cell, HGB: hemoglobin, Lym: lymphocyte, Neu: neutrophil, PLT: platelet) results of rats in various treatment groups on day 3 (n = 3). Data were presented as mean ± SD and statistical significance was analyzed via one-way ANOVA with Tukey's multiple comparison test.

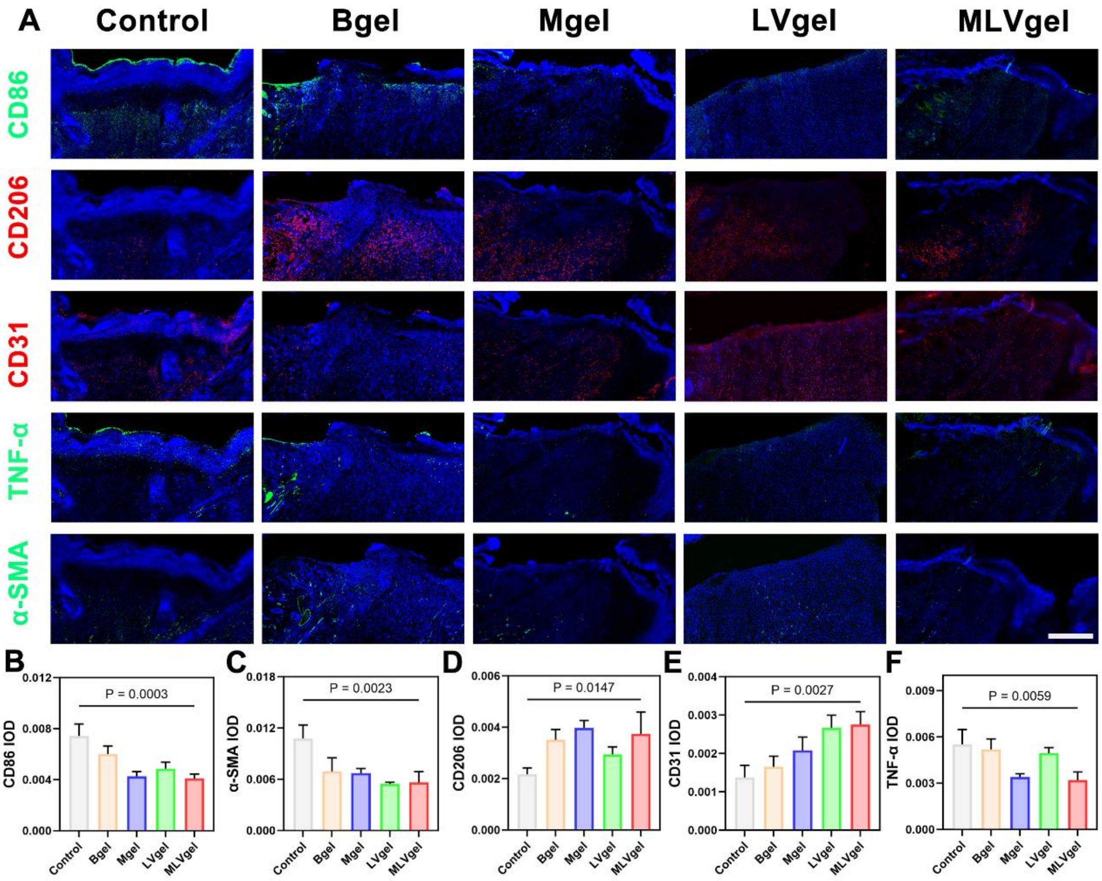

fig. S17. Analysis of immunofluorescence staining of infected wounds in rats on day 7 of infection. A) Representative immunofluorescence staining images about CD86, CD206, TNF-α, CD31 and α-SMA of rats wounds on day 7. Scale bar, 800μm. IOD analyze of B) CD86, C) CD206, D) TNF-α, E) CD31, F) α-SMA (*n* = 3). Data were presented as mean ± SD and statistical significance was analyzed via one-way ANOVA with Tukey's multiple comparison test.

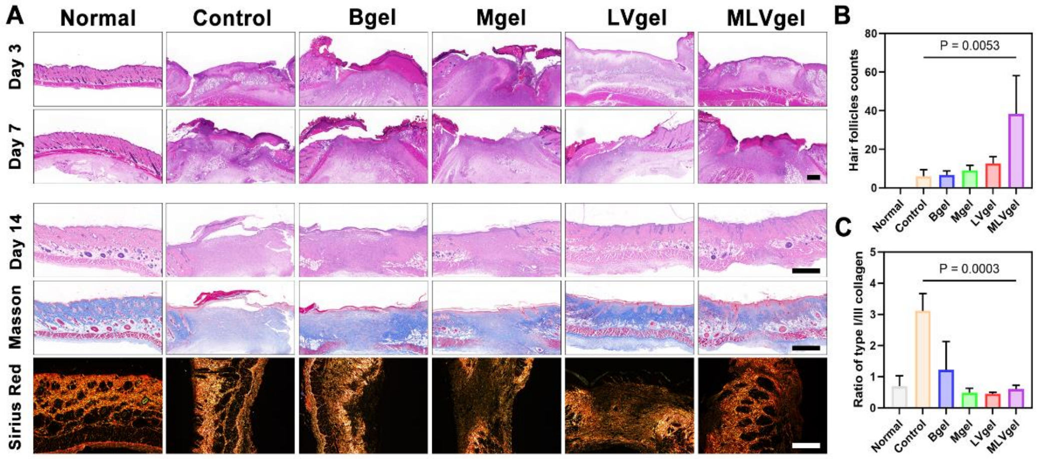

fig. S18. Evaluation of therapeutic effect on SD rat model of infected wound model. A) Representative H&E, Masson tricolor and Sirius Red staining images

of rat wound. Scale bar, 1mm. Quantitative analysis of B) new hair follicle and C) I/III collagen ratio (n = 3). Data are presented as mean ± SD and statistical significance was analyzed via one-way ANOVA with Tukey's multiple comparison test.

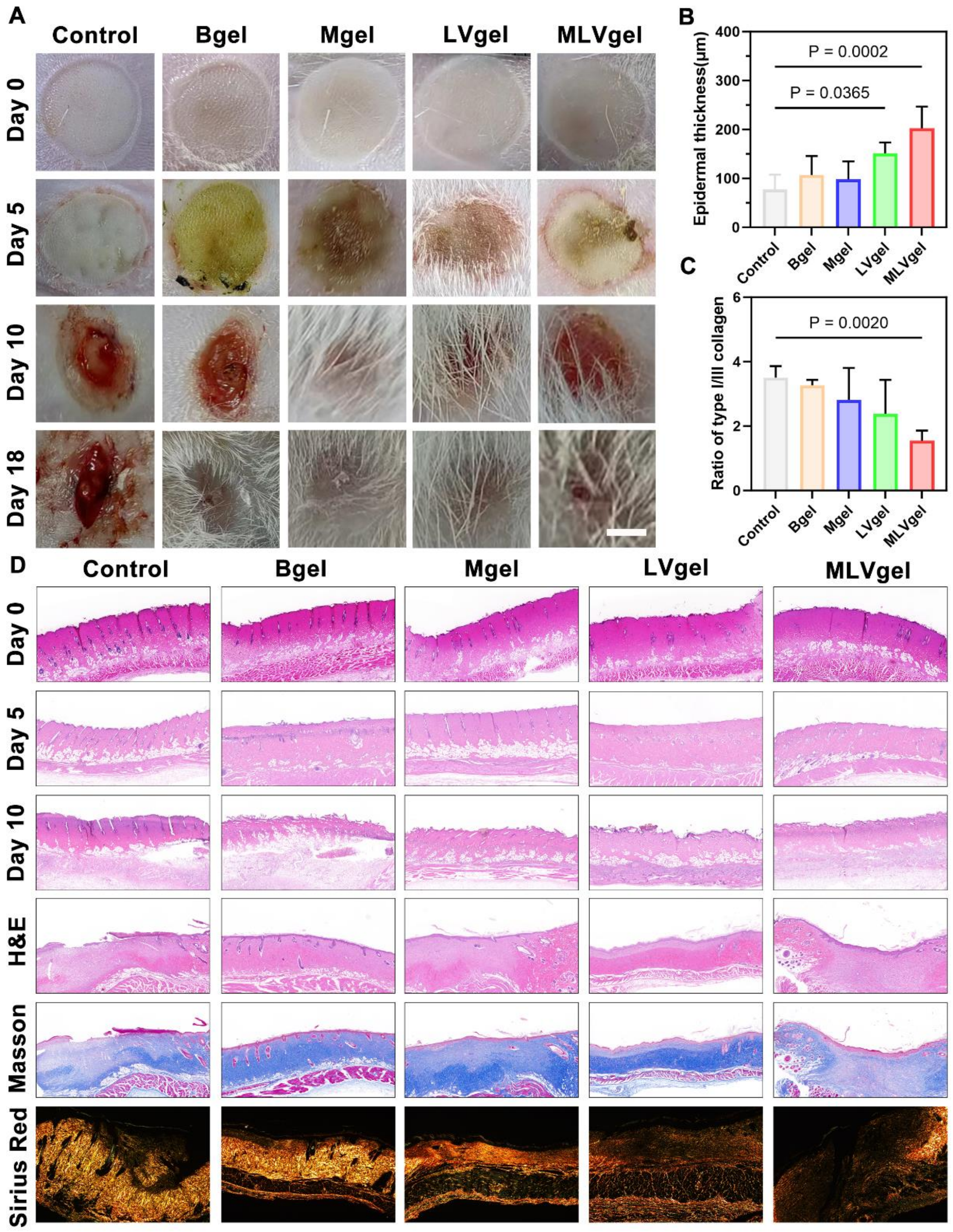

fig. S19. Evaluation of therapeutic effect on SD rat model of deep II burn. A) Wound photographs of SD rat deep II burn model in various treatment groups at different time points. Scale bar, 4cm. Quantitative analysis of B) epidermal thickness and C) I/III collagen ratio (n = 3). D) Representative H&E, Masson

tricolor and Sirius Red staining images of rat burn wound. Scale bar, 1mm. Data were presented as mean ± SD and statistical significance was analyzed via one-way ANOVA with Tukey's multiple comparison test.

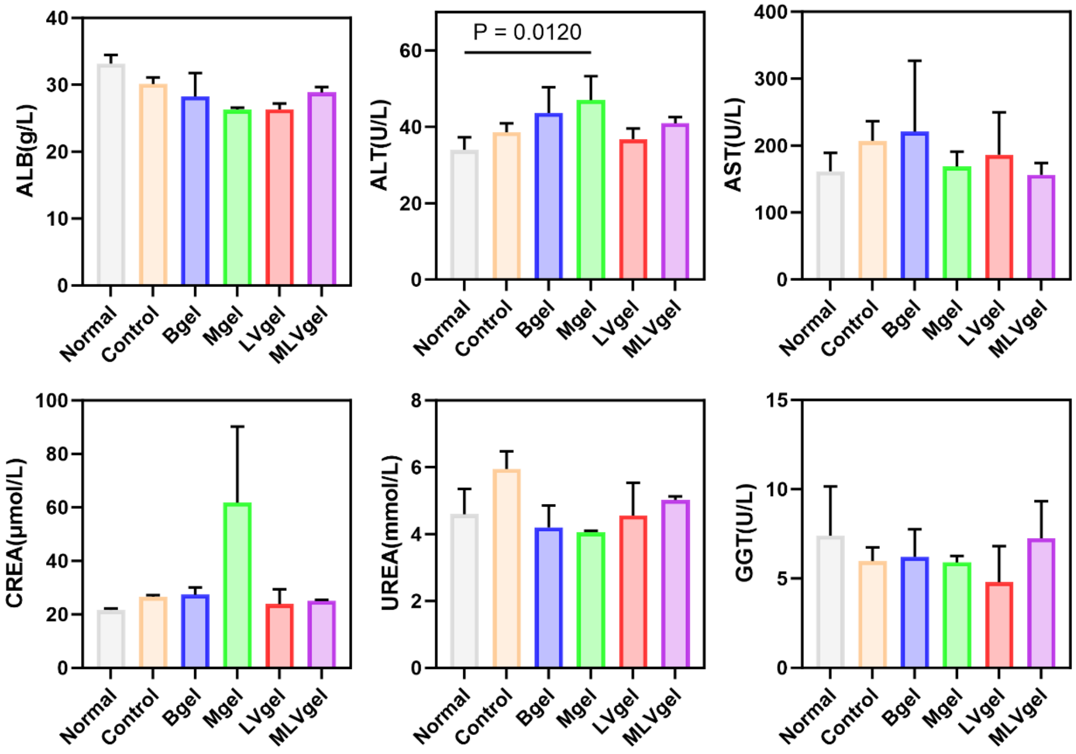

fig. S20. *In vivo* blood biochemistry analysis. Day 7 blood biochemistry results of rats in various treatment groups (*n* = 3). ALB: albumin, ALT: alanine transferase, AST: aspartate transferase, GGT: gamma-glutamyltransferase, CREA: creatinine. Data were presented as mean ± SD and statistical significance was analyzed via one-way ANOVA with Tukey's multiple comparison test.

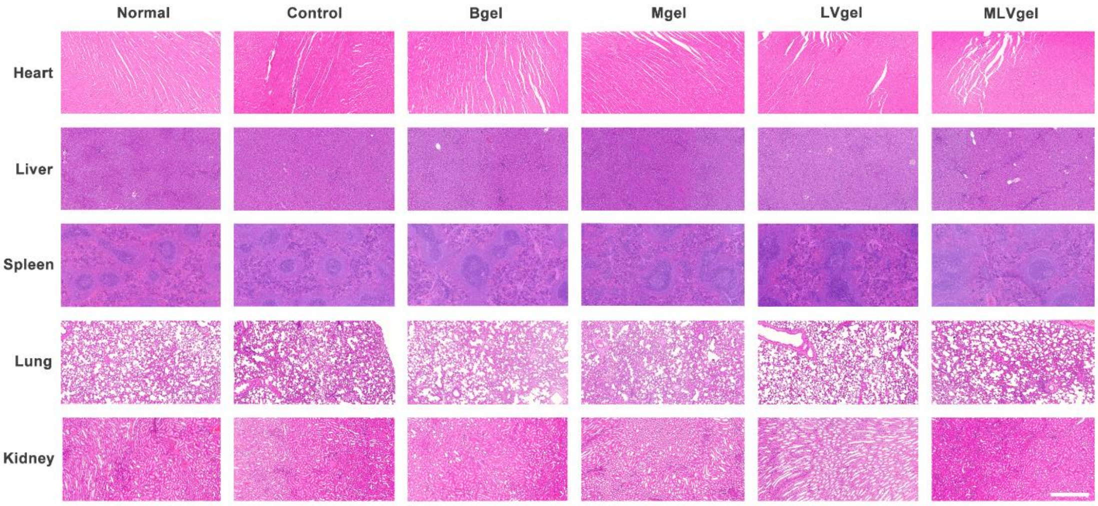

fig. S21. Biocompatibility of hydrogels. H&E staining images of major organs (heart, liver, spleen, lung, kidney) of rats following 14 days of various treatments. Scale bar, 100 μm.

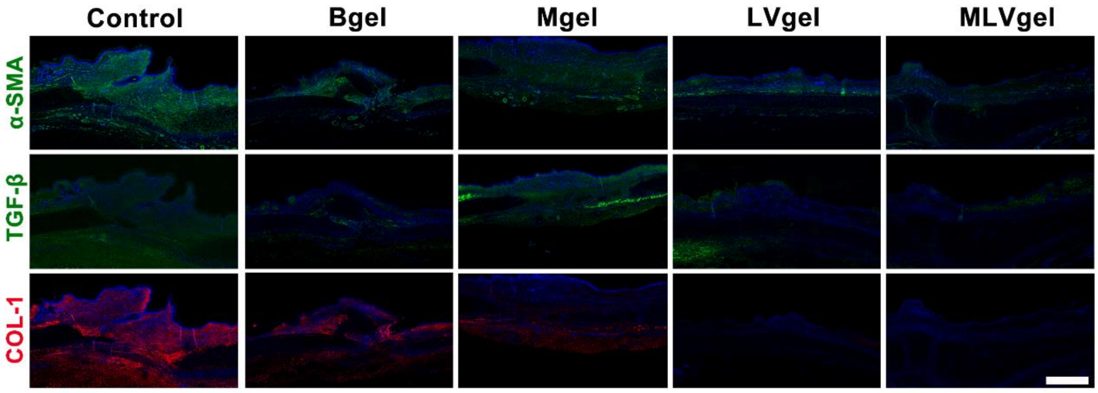

fig. S22. Analysis of immunofluorescence staining of rabbit ear scar model on day 28. Representative immunofluorescence staining images about α-SMA, TGF-β and COL-1 of wounds on day 28. Scale bar, 1mm.

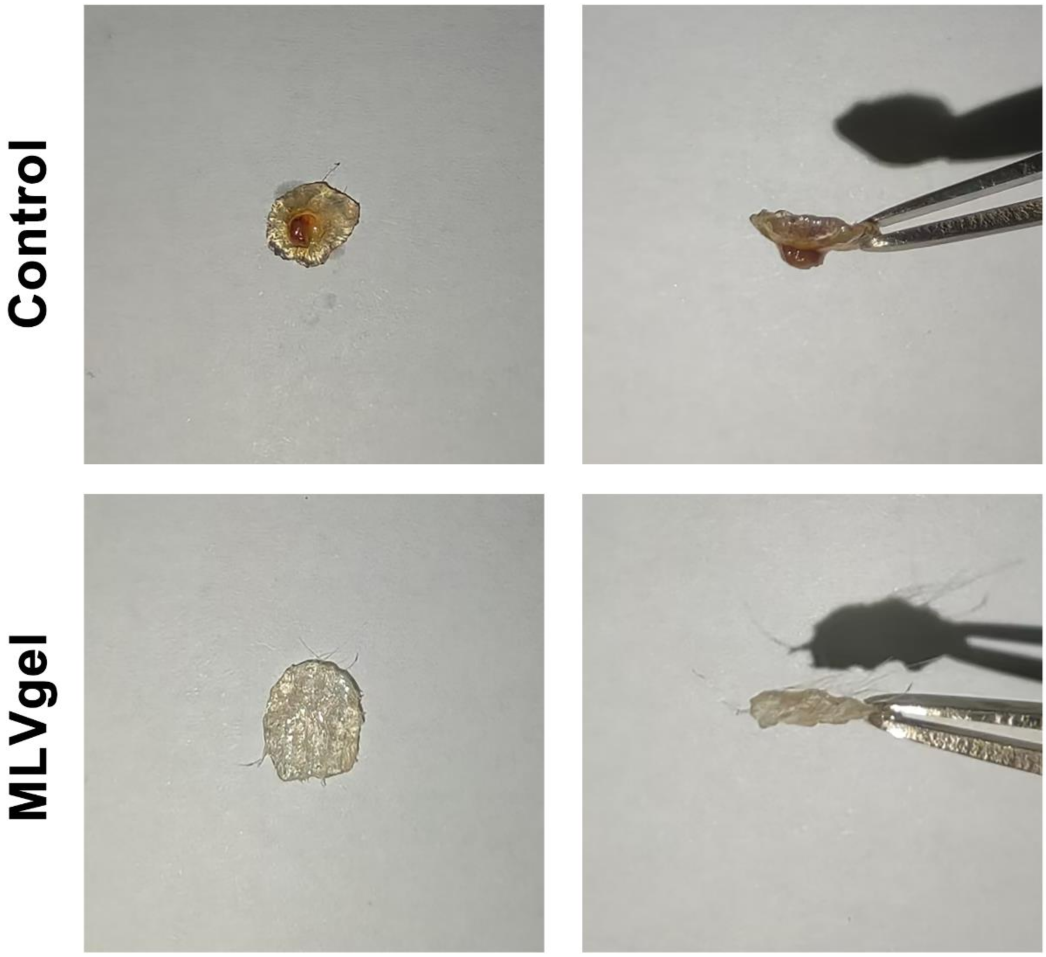

fig. S23. Representative image of transparent tissue.

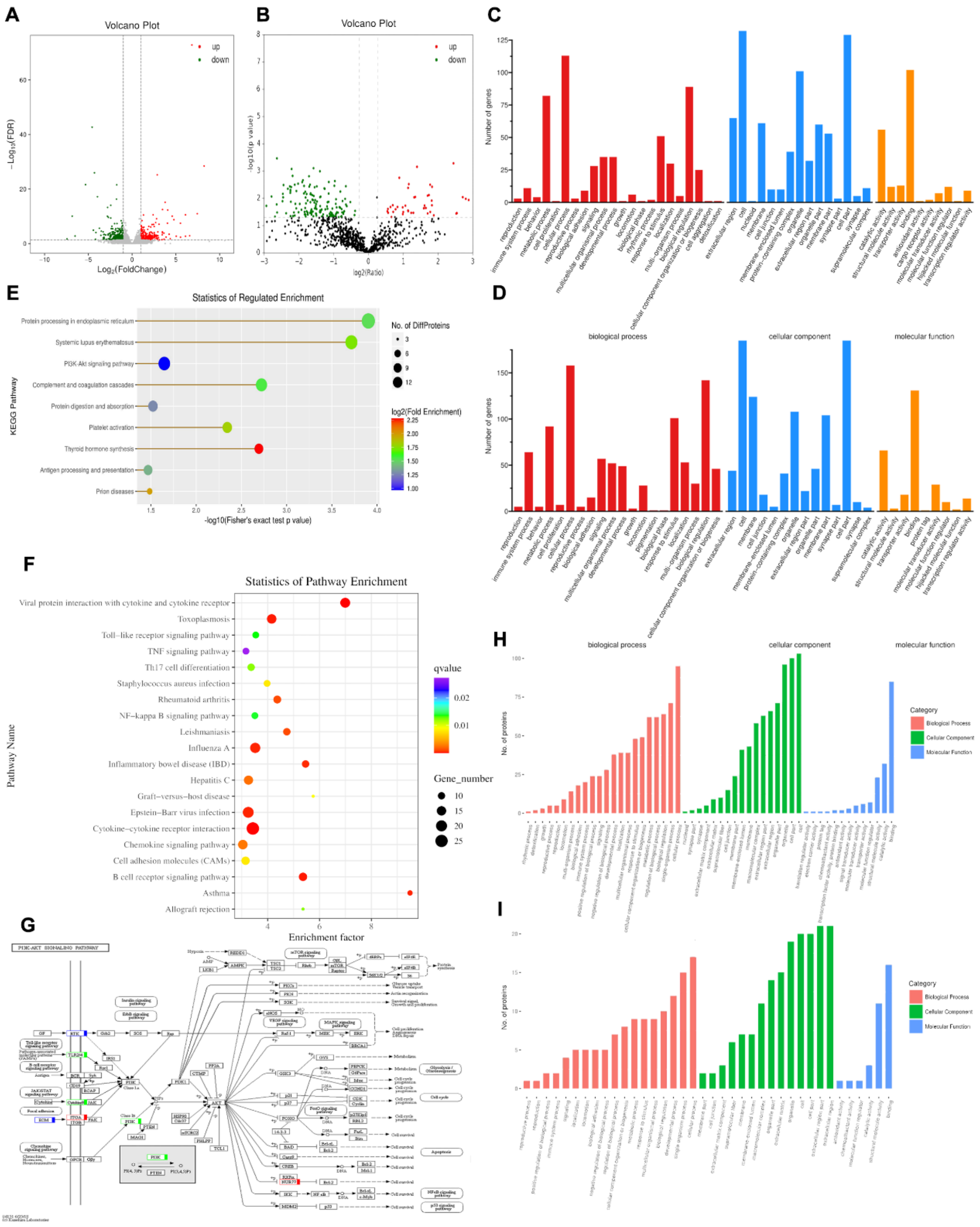

fig. S24. Transcriptomic and proteomic analysis of rabbit ear HS. A) Transcriptomic analysis of differentially expressed genes in volcano map. B) Proteomic analysis of differentially expressed proteins in volcano map. C) Up-regulation and D) down-regulation of GO analysis of differentially expressed genes. E), F) KEGG enrichment and G) representative path. H) Down-regulation

and I) Up-regulation of GO analysis of differentially expressed proteins. The size of the dot indicates the number of genes associated with the specified KEGG item, while the color of the dot represents the adjusted P value (P. adjust), calculated using a one-sided Fisher's exact test and Benjamini-Hochberg correction.

A

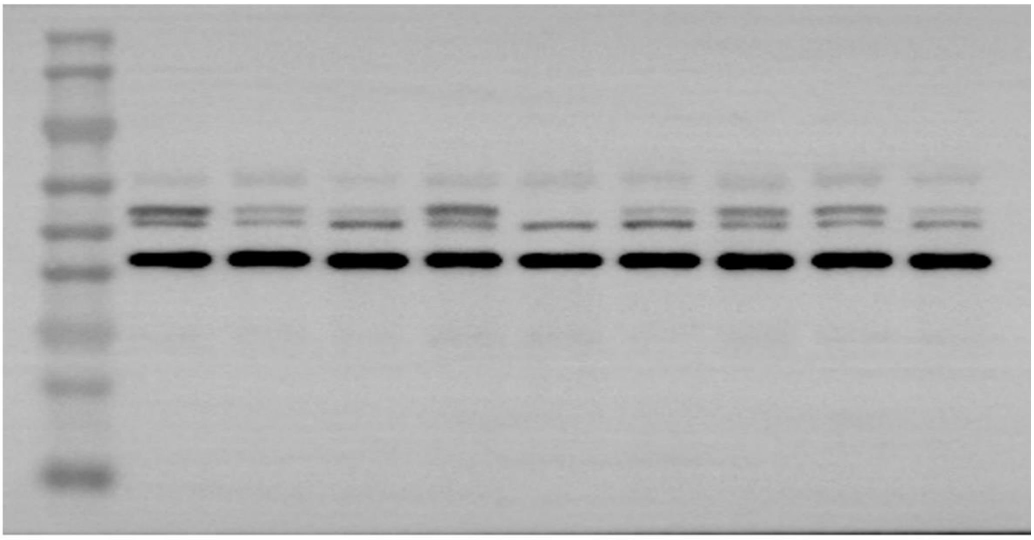

B

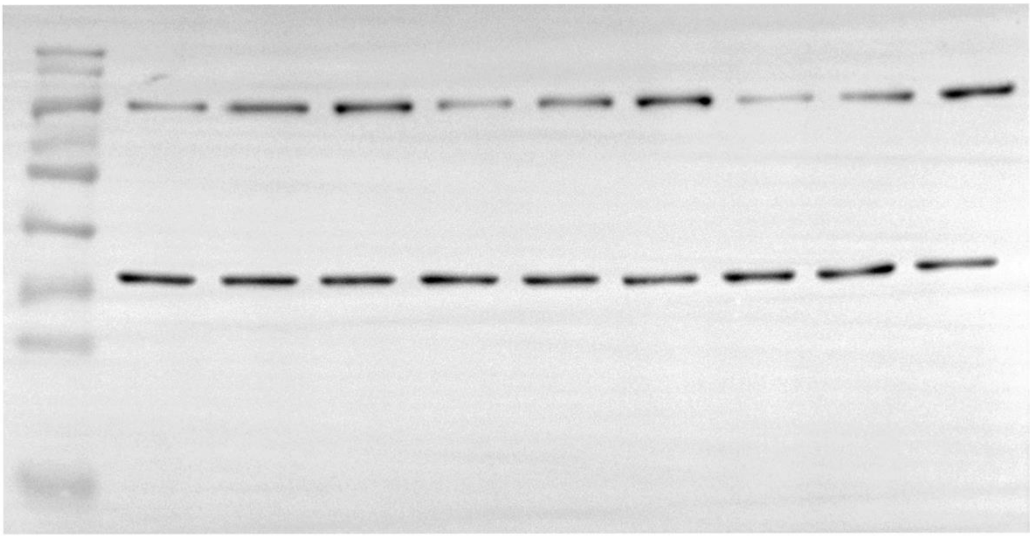

fig. S25. WB results for full membrane of relevant proteins. A) α-SMA/GAPDH, B) DCN/GAPDH.
